# Pathological mechanisms and therapeutic outlooks for arthrofibrosis

**DOI:** 10.1038/s41413-019-0047-x

**Published:** 2019-03-26

**Authors:** Kayley M. Usher, Sipin Zhu, Georgios Mavropalias, John A. Carrino, Jinmin Zhao, Jiake Xu

**Affiliations:** 1 School of Biomedical Sciences, University of Western Australia, Crawley, Western Australia Australia; 2 Department of Orthopaedics, The Second Affiliated Hospital and Yuying Children’s Hospital of Wenzhou Medical University, Wenzhou, Zhejiang China; 3 School of Medical and Health Sciences, Edith Cowan University, Joondalup, Western Australia Australia; 4 Hospital for Special Surgery, New York, NY USA; 5 Guangxi Key Laboratory of Regenerative Medicine, Guangxi Medical University, Nanning, Guangxi China; 6 Department of Orthopaedic Surgery, The First Affiliated Hospital of Guangxi Medical University, Nanning, Guangxi China

**Keywords:** Pathogenesis, Diseases, Bone quality and biomechanics

## Abstract

Arthrofibrosis is a fibrotic joint disorder that begins with an inflammatory reaction to insults such as injury, surgery and infection. Excessive extracellular matrix and adhesions contract pouches, bursae and tendons, cause pain and prevent a normal range of joint motion, with devastating consequences for patient quality of life. Arthrofibrosis affects people of all ages, with published rates varying. The risk factors and best management strategies are largely unknown due to a poor understanding of the pathology and lack of diagnostic biomarkers. However, current research into the pathogenesis of fibrosis in organs now informs the understanding of arthrofibrosis. The process begins when stress signals stimulate immune cells. The resulting cascade of cytokines and mediators drives fibroblasts to differentiate into myofibroblasts, which secrete fibrillar collagens and transforming growth factor-β (TGF-β). Positive feedback networks then dysregulate processes that normally terminate healing processes. We propose two subtypes of arthrofibrosis occur: active arthrofibrosis and residual arthrofibrosis. In the latter the fibrogenic processes have resolved but the joint remains stiff. The best therapeutic approach for each subtype may differ significantly. Treatment typically involves surgery, however, a pharmacological approach to correct dysregulated cell signalling could be more effective. Recent research shows that myofibroblasts are capable of reversing differentiation, and understanding the mechanisms of pathogenesis and resolution will be essential for the development of cell-based treatments. Therapies with significant promise are currently available, with more in development, including those that inhibit TGF-β signalling and epigenetic modifications. This review focuses on pathogenesis of sterile arthrofibrosis and therapeutic treatments.

## Arthrofibrosis: Drugs show promise for joint disease

“Stiff knee” or “frozen shoulder” syndrome, also known as arthrofibrosis, may come in two forms, active and residual,  which could benefit from different therapeutic interventions. A team led by Kayley Usher and Jiake Xu from the University of Western Australia in Crawley review the immune cells, signalling molecules and risk factors underpinning arthrofibrosis, a complication of surgery or trauma, in which scar tissue accumulates leading to painful restriction of motion in the shoulders, knees or other joints. The researchers propose the existence of two disease subtypes—one involving active scar formation, and one in which inflammatory processes have resolved—and they suggest each should be treated differently. Although surgery remains the most common intervention it may not be successful, and new research is highlighting the potential of pharmacological remedies, including those that block transforming growth factor-β signalling or target epigenetic modifications.

## Introduction

Arthrofibrosis is a fibrotic joint disorder characterised by excessive collagen production and adhesions that result in restricted joint motion and pain. It can occur in most joints,^1^ and is referred to by a number of names including frozen shoulder, adhesive capsulitis, joint contracture, stiff knee and stiff elbow. Sterile arthrofibrosis is typically caused by chronic or repetitive injury or surgery that leads to a dysregulated immune reaction and fibrosis in and/or around a joint^2^ to varying degrees. The fibrotic scar tissue that forms in the joint is known as extracellular matrix (ECM), and is primarily composed of collagen. Although the term ECM includes a wide variety of biological components we use this established terminology when discussing fibrotic scar tissue. This forms adhesions within joint capsules and contracts tendons and bursa around the joint,^3^ causing the loss of joint flexion and/or extension. In addition, scarred bursa may impinge into the joint causing more inflammation. Together with reduced range of motion (ROM), pain and varying amounts of swelling are commonly reported by patients. Arthrofibrosis affects people of all ages, although it is rare in children.^4^

Arthrofibrosis frequently causes significant disability; however, the nature of the disability depends on the joint affected and disease severity. When arthrofibrosis affects the knee symptoms become intensified during walking and standing, and the condition is frequently more debilitating than the original injury or degenerative condition.^5^ Even a small loss of knee extension of 5° creates difficulties in walking while a loss of flexion creates problems with stair climbing, sitting, getting in and out of chairs^6^ and cars and driving. Papers sometimes state that arthrofibrosis is a “frustrating” or “disappointing” problem for both surgeon and patient,^7–11^ however, these descriptions do not adequately describe the effects that arthrofibrosis has on patients’ lives. Patients frequently suffer constant pain, severe limitations on physical activity and difficulty sleeping, sitting and weight bearing.^12^ These symptoms may lead to the loss of job/career and difficulty socialising and performing daily living tasks, negatively impacting physical and emotional well-being.

On a cellular level arthrofibrosis is characterised by upregulated myofibroblast proliferation with reduced apoptosis, adhesions, aggressive synthesis of ECM that can fill and contract joint pouches and tissues and often also heterotrophic ossification.^1,13,14^ Although ECM is necessary for healing and wound repair, dysregulation of production and degradation leads to pathologic fibrosis.^1,15^ While there are relatively few studies into the pathogenesis and molecular biology of arthrofibrosis compared to other fibrotic diseases,^1^ there are common pathogenic pathways.^16–18^

This review highlights current progress in understanding the pathogenesis of sterile arthrofibrosis, focusing on arthrofibrosis of the knee to illustrate the condition. The regulation of inflammation, myofibroblast proliferation and survival and ECM production involves a highly complex array of mediators, cell types, receptors and interactions. A detailed explanation of all of these factors is beyond the scope of this review; therefore, we present a summary of the important cytokines and mediators involved in the condition. In addition this review examines currently available medications and developing pharmacological therapies that hold significant promise in the treatment of arthrofibrosis.

## Characterisation and classification of arthrofibrosis

**Fig. 1:**
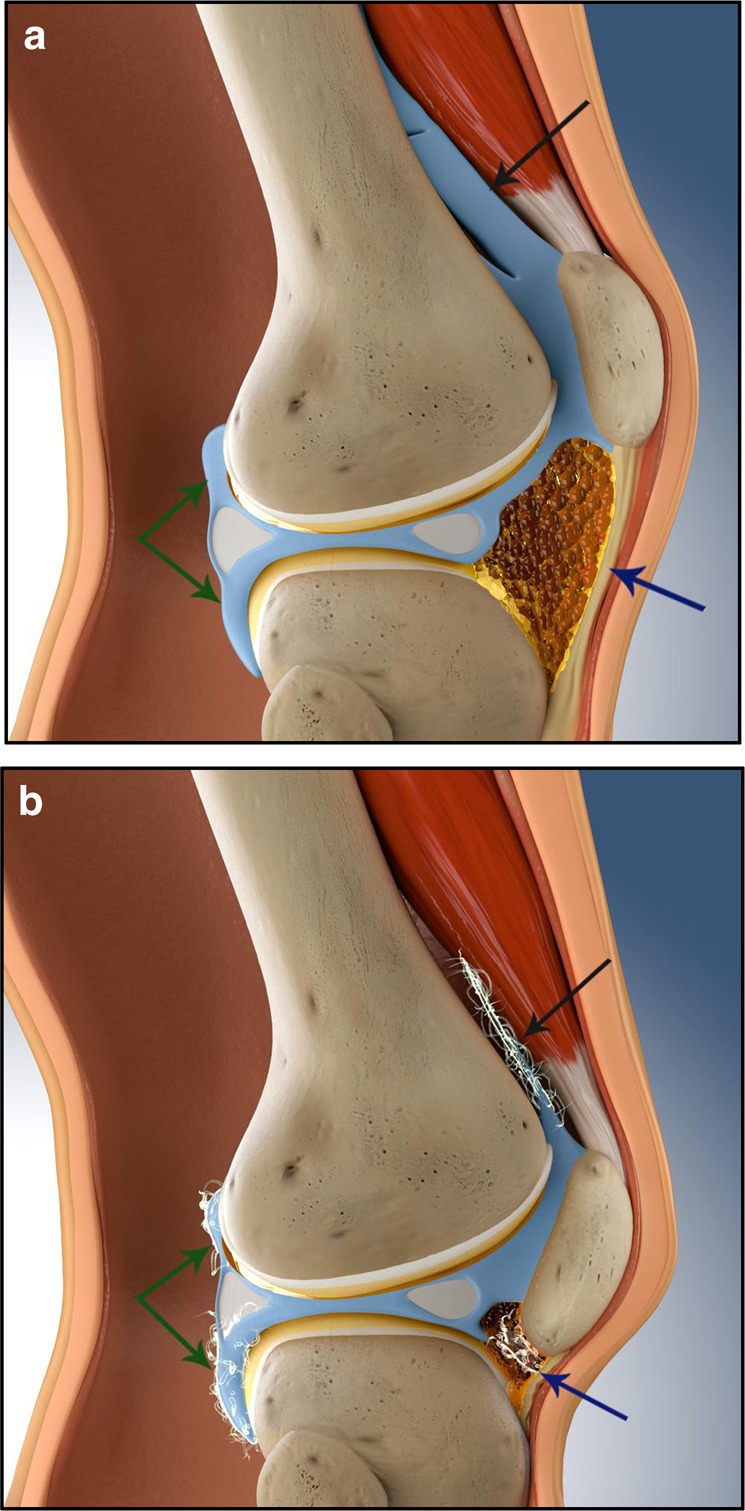
**a** Side view cross-section showing a healthy knee. **b** A knee with generalised arthrofibrosis. Major areas that are affected by arthrofibrosis are indicated. Black arrow = suprapatellar pouch. In “**b**” adhesions have pulled the walls of the pouch together with extracellular matrix (ECM) contracting the space and preventing normal movement. Green arrows = posterior capsule. In “**b**” scar tissue has contracted the folds of the posterior capsule, tightening them and affecting movement. The normal gutters at the side of the joint and the other bursae can also be affected. Blue arrow = anterior interval and infrapatellar bursa. In “**b**” inflammation and scar tissue has contracted the anterior interval and pulled the patella downwards, resulting in patella infera (baja). The patellar tendon adheres to the anterior interval and shortens, restricting movement

Although arthrofibrosis is often attributed to surgery, it can be caused by injury alone.^19^ This may be particularly true for shoulder arthrofibrosis (frozen shoulder), where the cause is often not known,^20^ but which may result from repeated small injuries over time, or damaged structures that place ongoing stress on the joint.^21^ The extent of involvement of the joint varies greatly. The formation of ECM may be localised, for example, cyclops lesions on tendons or generalised to involve much of the joint^6,12^ (Fig. 1). In knees the suprapatellar pouch, anterior interval, intercondylar notch, medial and lateral gutters, posterior capsule and infrapatellar fat pad (IFP or Hoffa’s fat pad), may all be affected,^6^ with symptoms varying depending on the location and extent of the ECM and adhesions, but typically involving loss of flexion and/or extension (see above).

**Fig. 2:**
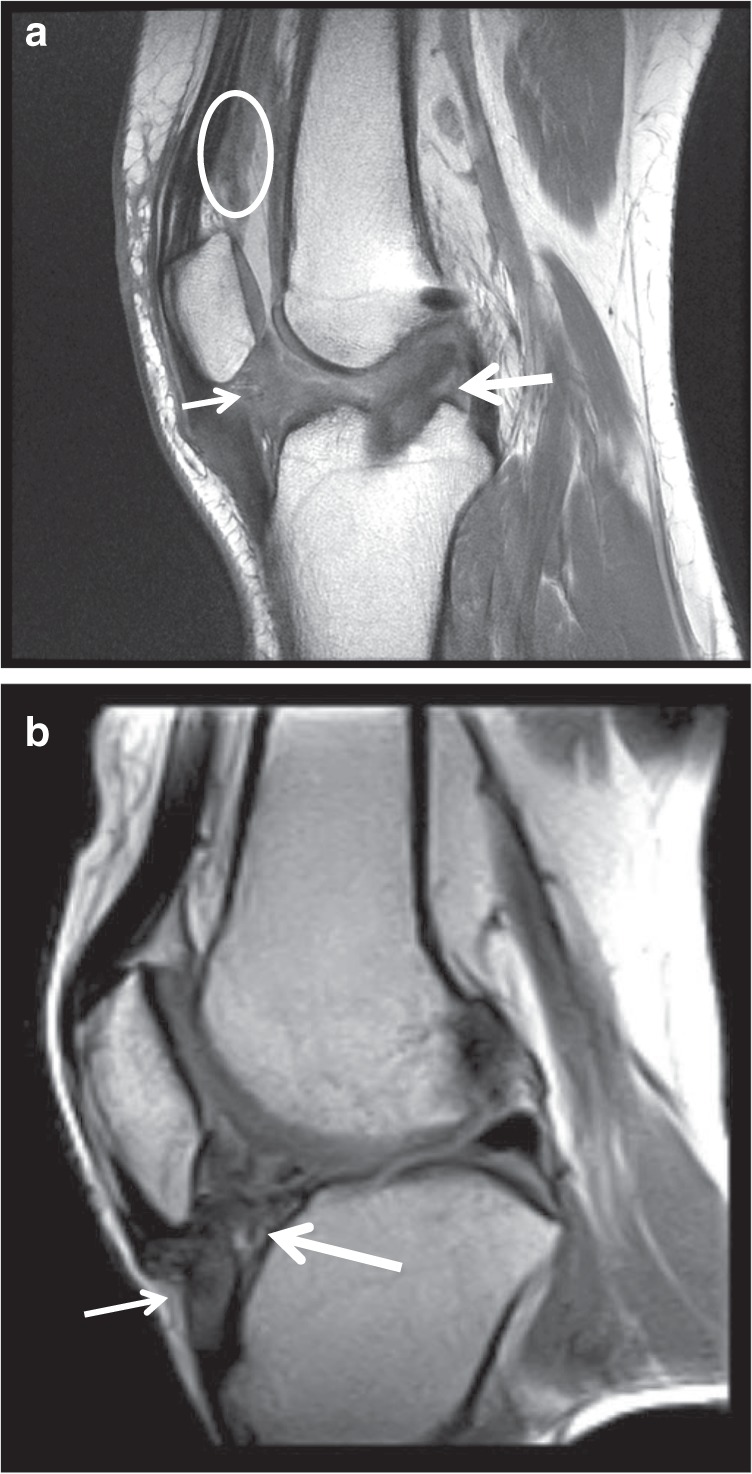
**a** Sagittal fast spin echo intermediate-weighted image of a 33-year-old woman with clinical stiffness following anterior cruciate ligament (ACL) reconstruction, showing scarring of the synovium around the ACL reconstruction (long arrow) as well as the central portion of the deep infrapatellar fat pad (short arrow) and the lining of the suprapatellar recess (oval). **b** Magnetic resonance imaging of the knee of a 49-year-old male with clinical stiffness 2 months following a meniscus operation, showing deep infrapatellar fat pad scarring (long arrow) and shortening of the patella tendon (short arrow) with resultant patella infera (abnormally low lying patella)

When the posterior capsule is affected contracture of ECM often prevents full extension of the leg, causing abnormal gait.^3^ ECM around the IFP causes patella infera (also called patella baja, Fig. 2). Shortening of the patellar tendon also contributes to this, leading to patellofemoral pain^22,23^ and often osteoarthritis (OA) at a later stage. The IFP may become fibrotic and impinge in the joint when the knee is flexed, creating further inflammation and fibrosis, loss of flexion and pain.^24^ The IFP is a store of immune cells that secrete inflammatory cytokines under stressful conditions^25^ (see “Risk assessment”), and can fill with ECM when adipose cells transform into fibrous tissue.^26^

The causes of arthrofibrosis are poorly understood,^27^ and explanations frequently depend on the training of authors. Shoulder arthrofibrosis has been recognised as an inflammatory condition for some time,^28^ however, orthopaedic surgeons specialising in knees have traditionally cited physical/mechanical causes such as poor surgical technique and non-compliance of patients in rehabilitation (for example,^7,9,29,30^). Nonetheless, the role that inflammation plays in arthrofibrosis is increasingly being recognised by the surgical community.^6,12,31^ Studies by immunologists and rheumatologists demonstrate that dysregulation of the immune system and wound healing processes, including inflammatory chemokines, cytokines and proteins, leads to fibrosis^18^ following an insult such as surgery. Indeed, surgery to treat anterior cruciate ligament (ACL) injury has been associated with a significantly higher risk of arthrofibrosis than conservative management.^29^ Immobilisation is also frequently cited as a contributing factor.^14,19^

Understanding arthrofibrosis, its causes, rates of occurrence and the success or failure of treatments has been complicated because the condition was poorly defined.^32^ Definitions have varied widely and are sometimes subjective, as are measures of treatment outcomes.^33^ Recently, an international panel of experts from multiple medical disciplines developed a consensus definition and classification of knee arthrofibrosis, which stated “post-operative fibrosis of the knee was defined as a limited ROM in extension and/or flexion”, measured by active flexion and extension, which was not caused by infection of other specific causes.^32^ Mild, moderate and severe arthrofibrosis was classified as flexion range of 90°–100°, 70°–89°, and less than 70°, respectively, and/or a loss of extension of 5°–10°, 11°–20° and more than 20^o^, respectively.^32^ The presence of pain was acknowledged as being an important aspect of the condition. This consensus definition should assist arthrofibrosis research and should be widely applied.

The Shelbourne classification^34^ has been widely used for knee arthrofibrosis in the past, but was developed from patients with arthrofibrosis arising from ACL reconstruction. Using these criteria a diagnosis of arthrofibrosis requires a loss of extension, excluding many patients with debilitating arthrofibrosis that have pain and a loss of flexion but not a loss of extension. For example, a recent case report identified a young woman with arthrofibrosis who had only minimal loss of ROM, but considerable pain, inflammation and disability.^12^ The presence of excessive ECM was confirmed by arthroscopy.

It is sometimes stated that arthrofibrosis is a rare complication of surgery^29^; however, some authors describe the condition as a common complication of total knee replacement (TKR) and ACL reconstruction surgeries.^8,33,35–37^ Estimates of the rates of arthrofibrosis following ACL reconstruction range from 2% to 35%,^22,29^ and after TKR between 0.2% and 10%^38^ with others reporting rates up to 15% (ref.^32^ and references within).^39^ One large study of TKRs in more than 64 000 patients in the US found that rates of arthrofibrosis for which revision surgery was performed was 0.2%.^38^ However, Abdul et al. reported post-TKR rates of arthrofibrosis of between 3% and 10%,^40^ and rates of 4%^41^ and 12%^42^ have been reported, with one review paper citing rates of stiffness from 8% to 60% following a TKR.^36^

In a study by Werner et al.,^5^ all surgeries in a national sample of specific cohorts for non-TKR knee surgeries were investigated. Rates of arthrofibrosis requiring a manipulation under anaesthesia (MUA) or arthroscopy within 6 months of the initial surgery were up to 8%. This study showed that rates of arthrofibrosis requiring surgical treatment were significantly higher for ACL reconstruction compared to meniscectomy and microfracture.^5^ However, even exploratory arthroscopies are capable of causing arthrofibrosis.^12^

While some of the confusion about the rates of post-operative arthrofibrosis are due to the lack of an agreed definition,^32^ other factors most likely come into play too. Papers may not reflect the true rates of arthrofibrosis^29^ due to reporting bias. Actual rates of arthrofibrosis following surgery are likely to be higher than the reported rates, since patients may not be treated surgically.^5^ Registries of joint replacement outcomes do not include arthrofibrosis unless the patient undergoes a surgical procedure to exchange or remove prostheses,^32^ and the incidence of untreated arthrofibrosis is unknown.

Arthrofibrosis is a form of fibrosis^43^ and common pathogenic pathways occur in fibrosis of organs and tissues.^15,17,44,45^ However, specialised cell types in some organs may have organ-specific influences.^43^ In fibrosis myofibroblasts are activated and dysregulated as a result of inflammation,^46^ and inflammatory cytokines are known to upregulate the factors that induce arthrofibrosis.^43^

Despite the increasing use of preventative measures after surgery, it appears that arthrofibrosis rates have remained relatively constant.^29^ A lack of an understanding of the role that inflammation plays in arthrofibrosis can lead to overly aggressive physical therapy programmes, with papers frequently recommending “aggressive” physical therapy as soon as possible after surgery.^7,42,47,48^ However, aggressive exercise can initiate or worsen arthrofibrosis^32,48^ because exercise triggers an inflammatory response^49^ including an increase in inflammatory cytokines, collagen production and TGF-β,^50,51^ factors that are dysregulated in fibrosis (see below). Some patients on international knee forums report that their symptoms either began or became significantly worse after they were instructed to “push through the pain” during rehabilitation, or performed more strenuous exercise.

## Two “types” of arthrofibrosis?

Pain and some degree of inflammation are recognised symptoms of arthrofibrosis,^32^ yet some papers on knee arthrofibrosis only discuss “stiffness” as a symptom, for example,^8,11,52^ and either specify a painless joint,^7^ or do not mention pain and inflammation at all. We suggest that what is termed “arthrofibrosis” may be two different conditions, (1) an active condition in which ECM formation and inflammation are continuous processes driven by positive feedback loops and (2) residual arthrofibrosis, in which the joint has limited ROM due to existing ECM, but the active inflammatory and ECM deposition phases have resolved. The presence of the inflammatory cytokines tumour necrosis factor alpha (TNF-α) and interleukin-1β (IL-1β) in acute pulmonary fibrotic tissue, but not in older fibrotic tissue,^53^ suggests one way in which active and residual fibrosis may differ, and an explanation in part for differing pain levels between the two arthrofibrosis groups, but research is lacking.

Misdiagnoses may complicate the understanding of arthrofibrosis. For example, Pujol et al.^35^ describe two types of patients with arthrofibrosis, those with swelling and pain in addition to loss of ROM, and those with primarily a loss of ROM. The first group of patients is described as having complex regional pain syndrome (CRPS), a type of neuropathic pain caused by nerve damage, and the authors recognise that this group of patients should not be operated on. However, there are no specific diagnostic tests for CRPS, and no clinical features that identify it.^54,55^ Consequently, the diagnosis of CRPS is made in the absence of other explanations for pain and swelling, and it remains a controversial diagnosis.^54,55^

Without publically available blood tests for arthrofibrosis, it seems likely that many patients that have been diagnosed with CRPS do in fact have active arthrofibrosis and a dysregulated inflammatory response. Indeed, a significant majority of patients diagnosed with CPRS type 1 have muscle weakness or limited ROM (ref.^55^ and references within). It is nonetheless worth recognising that inflammatory cytokines sensitise the peripheral and central nervous system leading to persistent pain in the presence of chronic low-grade inflammation.^56^

Indeed, under these conditions it is thought that persistent synthesis of substance P, a known pain sensitiser and activator of mast cells and fibroblasts, occurs, and creates a positive feedback loop.^14^ In support of this, an increased ratio of sensory nerves (expressing substance P) to sympathetic nerves was found in tissue from arthrofibrotic knees.^57^ Also of note is the fact that chronic low grade inflammation frequently does not have obvious physical signs or markers in the blood,^56^ but can nonetheless play a role in active arthrofibrosis.

More research is needed to understand the difference between active and residual arthrofibrosis, as the response of patients within these groups to surgery and exercise may be significantly different. In support of this, Panni et al.^7^ report that painful stiff knees do not respond well to arthroscopic surgery to lyse adhesions, and Babis et al.^27^ report that surgery to treat arthrofibrosis in TKR patients resulted in worse outcomes for pain in all patients, with some also losing flexion. Surgical lysis of fibrotic material is the standard treatment for arthrofibrosis, however, surgery stimulates wound healing processes, including ECM proliferation, and is associated with increased inflammation.^58^ In addition, immune system memory and/or feedback processes that may be occurring in a patient with active arthrofibrosis may be further stimulated by surgery. It is known that re-occurrence is frequent after the removal of ECM in some conditions.^15^

Possible parallels with active and residual knee arthrofibrosis can be found in shoulder arthrofibrosis, in which pain may resolve with time or remain together with ROM limitations,^28^ and in other fibrotic diseases. There are several fibrotic diseases of the lungs, including simple pneumoconiosis, in which fibrosis begins and stops, and progressive massive fibrosis, in which extensive fibrosis progresses until fatal.^59^ Simple pneumoconiosis can turn into progressive massive fibrosis if exposure to dust and inflammation continues. Liver fibrosis is another possible parallel, as it can sometimes be stopped and even reversed^60^ using anti-inflammatory or anti-viral medications, but can turn into active, progressive fibrosis.^61^ Active fibrosis results from a switch from an initial Th1 inflammatory cell response to a Th2 cell response with prolonged exposure to an inflammatory stimulus. While this switch helps to control the damage caused by immune cells and promotes healing, it also activates collagen deposition and fibrosis.^62^

## Genders differences in rates of arthrofibrosis

Women have been reported to be more likely to develop arthrofibrosis than men,^21,63^ with studies citing rates 2.5–2.8 times higher,^29,64^ although others have not found a gender difference.^33,38^ It has been suggested that the higher rates of arthrofibrosis in women may be due to psychological differences between the genders and that women may be less active post-operatively, may not perform rehabilitation as well as men, may seek more medical interventions, and have “different” pain tolerance than men.^29^ But Hemsley^65^ found no differences in pain perception or pain reflex between patients at 6 weeks post-ACL reconstruction surgery, almost half of whom did not recover full ROM.

However, it is well established that the genders differ in their immunological responses, with 80% of autoimmune disease occurring in women.^66^ Being female is also a risk factor for OA,^38,67^ with more women undergoing TKR than men, despite women having a greater unmet need for this surgery.^68^ Recent research shows that OA is initiated and progressed by inflammation (see below in Risk factors), and that patients with OA have high levels of inflammatory cytokines in the knee.^58^

The gender difference in inflammatory responses is due to both genes and hormones. Women have stronger innate and adaptive immune responses than men, leading to increased rates of inflammatory and autoimmune diseases.^66^ The corollary is that women have around half the risk of serious post-surgical septic infection,^69^ possibly because oestrogen upregulates pro-inflammatory cytokines including IL-1 and IL-6.^70^ Transforming growth factor β (TGF-β), the primary driver of fibrosis, is also upregulated and activated by progesterone and oestrogen,^71^ driving an increase in Treg cells at ovulation.^72^ Because immune system dysfunction and acute inflammation cause fibrosis,^2^ the higher rates of arthrofibrosis in women is likely due to these immunological differences between the genders.

## Risk factors for arthrofibrosis

There are no established methods for determining the risk of developing arthrofibrosis following surgery. However, by understanding the pathology of the condition, it may be possible to prevent or successfully treat arthrofibrosis,^13,42^ and a number of factors are known to be involved (Table 1). Early onset OA may be a risk factor/indicator for developing arthrofibrosis after injury or surgery. OA is associated with inflammation,^73–76^ and the inflammatory cytokines IL-6 and TNF-α are upregulated in OA synovial fluid.^67,74^ Importantly, in a study by Remst et al. over half of patients with OA were found to have fibrosis of the synovium,^43^ and other studies have also found an association between OA and fibrosis.^75,76^

**Table 1:** The stages of pathogenesis of sterile arthrofibrosis of the knee with corresponding clinical features, risk factors and current managements

Pathogenesis	Clinical features	Risk factors	Current management
Inflammatory response, upregulated TGF-β	Pain, redness and swelling	Surgery or injury	•Elevation and icing •Corticosteroids •Aspirin
Proliferation of myofibroblasts and ECM production	Stiffness and restricted range of motion	Surgery or injury	
Dysregulation of inflammation and TGF-β signalling, excessive ECM in and around joint, adhesions and contractions. Epigenetic alterations	Persistent pain and restricted ROM, with typically mild swelling. Further ECM production and contractions of soft tissues, abnormal gait	•Previous surgeries •Mutations causing excessive TGF-β or inflammation •Female gender? •Early onset OA •Inflammatory and autoimmune diseases	•Daily CPM •Exercise rehabilitation •Control of inflammation •MUA •Surgery to lyse adhesions and debride ECM

*ECM* extracellular matrix, *TGF-β* transforming growth factor β, *ROM* range of motion, *OA* osteoarthritis, *CPM* continuous passive motion machine, *MUA* manipulation under anaesthesia

This link with arthrofibrosis is likely due to over-expression of TGF-β, a well-known initiator of fibrosis (see below) that is also implicated in the development of OA when expressed at high levels in subchondral bone and synovial cells.^77^ TGF-β levels were higher in subchondral bone of patients with OA compared to healthy controls, and appeared to lead to increased blood vessel formation, bone resorption and stress on articular cartilage.^78^ In support of this, high levels of TGF-β induced in rats and mice have led to OA-like lesions.^78,79^

This suggests that a pro-inflammatory, pro-fibrosis scene exists for patients with early onset OA. The high numbers of fibroblasts in knee synovium can drive inflammation^67^ and become further activated following surgery. In addition, patients with OA have a more pro-inflammatory lipid profile in the IFP than individuals with healthy joints.^25^The bursa around the knee, particularly the IFP, produce and store inflammatory cytokines^26,58^ and immune cells, including macrophages, T cells, B cells and mast cells that can be locally activated by an insult to secrete inflammatory cytokines, particularly TNF-α and IL-6.^25,80^ Macrophages have been detected in the IFP at 20 weeks post-ACL reconstruction surgery,^58^ and are known to play a key role in all stages arthrofibrosis.^81^

Injury prior to surgery is also a risk factor for arthrofibrosis. ACL tears have been demonstrated to increase the levels of IL-1β and TNF-α in synovial fluid, with levels increasing with the degree of damage and with time since injury.^82^ It has been suggested that higher levels of these cytokines are responsible for the later development of OA.^82^ TGF-β is also upregulated in the IFP at 2 weeks post-ACL reconstruction surgery,^58^ potentially contributing to the high rates of arthrofibrosis after this type of surgery. More than two previous surgeries are also a risk factor for post-operative arthrofibrosis,^11^ indicating that there is a potentiation or “memory” of each insult, as demonstrated in other fibrotic diseases.

In other surgery, such as TKR and reconstructive surgery using artificial ligaments, the implantation of a prosthesis triggers the formation of fibrotic tissue as the body attempts to encapsulate the foreign material.^83^ Implants such as screws that impinge on tissues also cause an inflammatory reaction,^84^ and may promote arthrofibrosis of TKRs that are not well fitted.

Other factors can also come into play. Childhood adversity such as neglect or abuse is associated with disease and disability later in life,^85^ causing higher Th17 cell numbers, a higher IL-6 response to stress, and autoimmune and inflammatory diseases.^86^ Depression and associated poor rehabilitation compliance are sometimes cited as causative factors for arthrofibrosis,^7^ however, it is interesting to note that depression is strongly associated with inflammation, and inflammation can cause depression.^87,88^ Therefore, it seems likely that the inflammatory processes associated with active arthrofibrosis cause depression.

Other risk factors include pre-existing inflammatory or autoimmune diseases, including type II diabetes,^20^ ankylosing spondylitis and rheumatoid arthritis.^7^ One study found that patients with diabetes mellitus had increased rates of arthrofibrosis after a TKR,^38^ possibly due to a pro-inflammatory physiology.

Biomarkers to assess the risk of developing post-surgical arthrofibrosis are urgently needed. In addition to pre-surgery applications, biomarkers could also be used post-operatively for all joint surgeries to monitor potential for developing arthrofibrosis, and following a diagnosis, to monitor the condition and its resolution. Such biomarkers will be essential for the development and testing of therapies.^89^ Ideally tests should be minimally invasive, for example, serum parameters and imaging, and applicable before surgery and during treatment to follow progress.^90^

## Genetic risk factors

Some patients may have a genetic predisposition for developing fibrosis,^91^ with a twin study finding there was a genetic component to shoulder arthrofibrosis.^92^ Because multiple biological pathways impact on the pathology of arthrofibrosis, it is likely that there are many types of mutations that can affect the risk of developing it, including mutations in the immune system, TGF-β signalling and genes involved in the synthesis or degradation of collagen. Skutek et al.^93^ found a possible link between some varieties of human leucocyte antigen and the risk of arthrofibrosis. The human leucocyte antigen complex is involved in immune system functioning.

People with mutations involving TGF-β production or signalling, which can result in excessive ECM formation,^94^ may be at particular risk of developing arthrofibrosis. One candidate condition is Aneurysms-OA Syndrome, now included under the name Loeys–Dietz syndrome, in which upregulation of TGF-β signalling causes early onset OA.^95–97^

## Pathogenesis of fibrosis

**Fig. 3:**
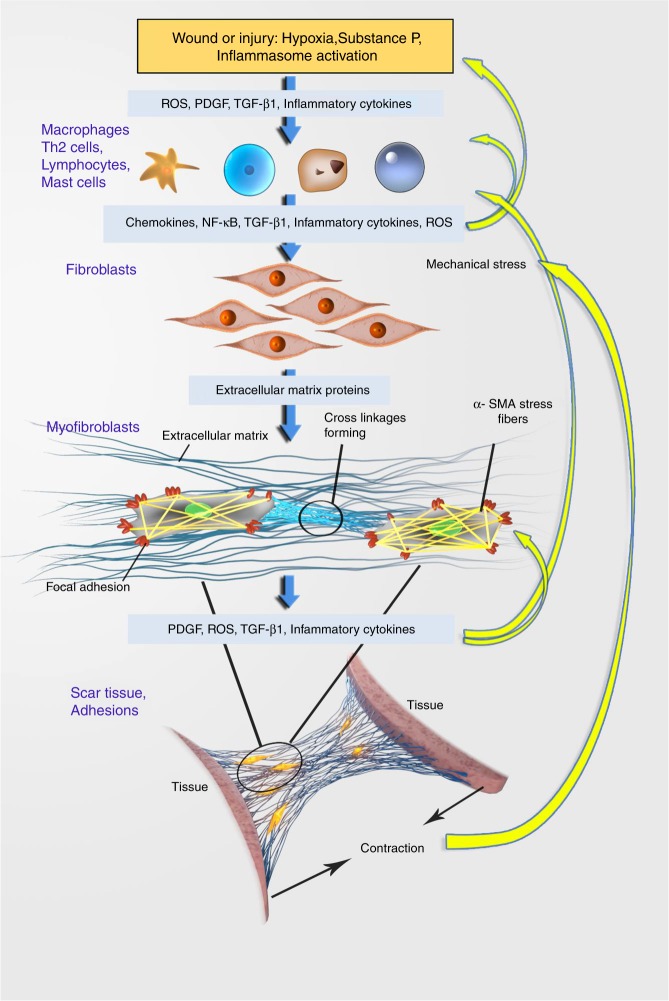
An insult such as surgery or injury causes hypoxia and activates inflammasomes in cells, resulting in the production of reactive oxygen species (ROS), platelet-derived growth factor (PDGF), transforming growth factor beta (TGF-β) and a range of inflammatory cytokines and mediators. These activate immune cells, causing more inflammation and a cascade of events that stimulates fibroblasts to differentiate into myofibroblasts, the key mediators of fibrosis. Dysregulation and positive feedback loops (curved yellow arrows) result in persistent pathological fibrosis. TGF-β plays a central role in the process, stimulating fibroblasts to proliferate and differentiate, and to increase their extracellular matrix (ECM) production. TGF-β also induces the production of ROS and regulates T cell differentiation and proliferation. Nuclear factor kB (NF-κB) produced by macrophages is activated by TGF-β, as well as many of the inflammatory cytokines induced by it. PDGF promotes the migration, proliferation and survival of myofibroblasts and upregulates TGF-β synthesis by fibroblasts. The production of IL-1β by macrophages further stimulates inflammasomes. Mechanical forces and stress also alter fibroblasts, causing them to differentiate into myofibroblasts. The fibres of α smooth muscle actin (α-SMA) inside myofibroblasts terminate with adhesion complexes on myofibroblast surfaces and attach to ECM and other cells, generating contractile forces. Over time the cross-linkages in the ECM and focal adhesions become more complex and further tissue contractions occur. Myofibroblasts resist apoptosis and are able to maintain themselves by secreting TGF-β

**Fig. 4:**
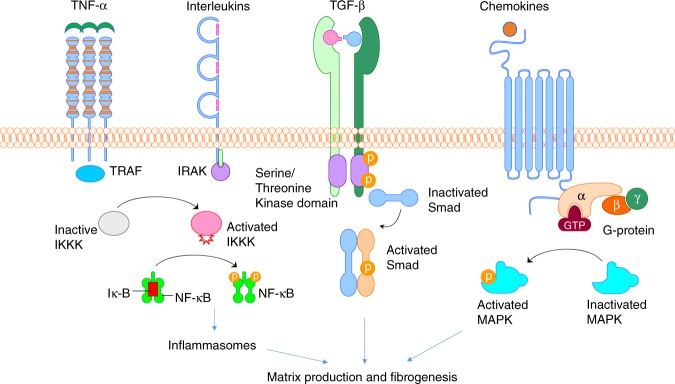
Four potential signal transduction pathways and their corresponding receptors associated with arthrofibrosis; including TNF-α, Interleukins (IL1, IL6, IL17, etc.), TGF-β and chemokines ligand-receptor superfamilies, which lead to activation of NF-κB, Smad, MAPK and multiple downstream gene transcriptions responsible for matrix production and fibrogenesis

There is little research into the cell biology and pathogenesis of arthrofibrosis. However, a wealth of organ fibrosis research provides important insights into the processes involved in arthrofibrosis, and is reviewed here. Fibrosis results from a complex dysregulation of innate and adaptive immunity that is involved in most chronic inflammatory diseases,^15,45,46^ and is a leading cause of mortality.^62^ Injury causes oxidative stress and an inflammatory response, inducing pro-inflammatory cytokines^98–100^ and TGF-β (Figs. 3 and 4).^101^ This leads to an increase in mast cells, macrophages and lymphocytes that promote fibroblast proliferation and reduced vascularisation.^13,62^

A lack of apoptosis and autophagy within fibrotic tissues have also been implicated in a number of fibrotic conditions, and may contribute to fibrotic tissue formation.^13^ Reduced autophagy leads to a build-up of defective mitochondria and oxidative stress.^102^ Immune cell signalling also stimulates an increase in reactive oxygen and nitrogen species (RONS)^13^ and positive feedback between macrophages and lymphocytes, leading to immune cell dysregulation.^62^ However, the severity of fibrosis is often not well correlated with the degree of inflammation,^62^ and low-level inflammation that persists over long periods also causes fibrosis.^46^

Pro-fibrotic cytokines are thought to cause an imbalance between ECM production and degradation, leading to excessive deposition of matrix proteins, which are both collagenous and noncollagenous.^15,45^ Collagen type I is the main constituent of ECM. It has high-tensile strength that prevents normal stretching, and in fibrosis there is a higher ratio of collagen type I to stretchy elastin, compared to healthy tissues.^103^ In addition to altered composition, fibrotic ECM has extensive cross-linking that makes it very difficult to degrade.^89,104^ In particular, levels of hydroxyallysine cross-linking is increased, and appears to lead to irreversible collagen accumulation^105^ together with other effects on cell signalling and ECM synthesis.^89^

The ECM that forms in fibrosis is largely cell-free, and serves as a conduit for immune cells, fibroblasts, nutrients and endothelial cells during angiogenesis. In addition to proteins the ground substance of the ECM is comprised of proteoglycans, and these bind and inhibit or enhance a range of growth factors, proteases, protease inhibitors and TGF-β (for review see ref. ^103^).

The inflammatory cytokines and mediators that trigger fibrosis, together with the cells that express them (see below), are essential components of a healthy immune system. Typically, inflammatory cytokines are downregulated after a period of time, but the continued presence of inflammatory cytokines and mediators can cause tissue to become pro-inflammatory and fibrosis may develop. The presence of one inflammatory cytokine causes the receptors for other cytokines to be made, sensitising cells to respond strongly.^106^ Repeated trauma and/or long-term inflammation can trigger epigenetic modifications and activation of myofibroblasts and matrix-related genes.^46,107,108^ Chronic inflammation may also result from a lack of bioactive lipid mediators (LMs) that causes deficient or non-existent resolution (see “resolvins”), or LMs that don’t have the required regulatory effects.^109^

Almost all types of immune cells are involved in fibrosis^110^ and the pathways are extremely complex. Consequently, a detailed discussion is beyond the scope of this review, however, we explore the major cell types and cytokines involved below.

### Myofibroblasts

Myofibroblasts are the key effector cells of fibrosis,^46,111^ remodelling the ECM, and depositing dense fibrotic collagen.^15,44,112,113^ Myofibroblasts also form cell-to-cell connections and connections between cells and ECM, creating contractile units and causing the contraction of surrounding tissues.^103,114–116^ In the presence of TGF-β myofibroblasts produce fibres of α-smooth muscle actin (α-SMA) together with collagen type 1 (for review see^115,117^). Over time the focal adhesions become more complex and further tissue contractions occur, together with extensive collagen cross-linking.^108^

Myofibroblasts are important in wound healing, however, they are not usually found in healthy tissue.^103^ They are derived from fibroblasts^115,116^ and a range of other cells^107,113^ that have differentiated in response to inflammatory cytokines such as TGF-β, IL-1β and IL-6. However, myofibroblasts also produce TGF-β, IL-1β, IL-6 and platelet-derived growth factor (PDGF), in addition to reactive oxygen species (ROS) and a range of paracrine signals that further activate a fibrotic response (for reveiw see ref. ^103^). Thus myofibroblasts resist apoptosis and are able to maintain themselves by secreting TGF-β^15^ and inflammatory cytokines, activating immune cells and further fibrosis. In addition, mechanical forces also alter the biochemical actions of fibroblasts, causing them to differentiate into myofibroblasts.^114^

During normal wound healing and resolution of inflammation some myofibroblasts become apoptotic, while others revert to the original cell type, however, the processes by which this occurs are not yet understood.^90^ In fibrosis epigenetic alterations in myofibroblasts increase the activity of inflammatory and pro-fibrotic genes^118^ (see below in Epigenetic alterations), and appear to serve as a type of memory of the insult.^108^ Myofibroblasts that have reverted back to fibroblasts are more likely to become re-activated when exposed to further insult.^60,90^ This has implications for repeated joint surgeries as fibrosis may resolve naturally and unnoticed, but the presence of reverted fibroblasts that serve as a store of pre-fibrotic cells may leave the patient susceptible to arthrofibrosis after subsequent surgeries, as discussed earlier. It is not known if the formation of ECM is common following surgery, only becoming apparent when normal function is compromised.

## Inflammatory cells and cell structures

A number of cell types contribute to the initiation and maintenance of chronic inflammation and fibrotic diseases, including macrophages, myofibroblasts and Th2 cells.^62^ In addition to these factors, protein complexes within the cytoplasm of cells called inflammasomes produce inflammatory cytokines, and which serve as a type of “memory” of insult (see below).

### Macrophages

Macrophages react to a diverse range of signals by secreting cytokines and chemokines, and are found in close association with myofibroblasts.^119^ They can be activated by TGF-β and can be important in fibrosis.^16^ Classically activated macrophages (M1) secrete inflammatory cytokines, including TNF-α, IL-1 and IL-6.^120^ M1 also promote the differentiation of Th17 cells, which are also pro-inflammatory (see below). However, M2 macrophages secrete anti-inflammatory cytokines, including IL-10 and IL-13, and are important in the resolution of inflammation.^120^

Recent research shows that distinct macrophage populations may control the initiation, maintenance and resolution of fibrosis.^81^ Macrophages are an important source of the pro-fibrosis mediators TGF-β, IL-1β and PDGF.^46^ PDGF promotes the migration, proliferation and survival of myofibroblasts,^121,122^ and upregulates TGF-β synthesis by fibroblasts.^123^ In addition, the production of IL-1β by macrophages can stimulate inflammasomes in the lung.^46^ Macrophages may be able to regulate ECM synthesis independently of TGF-β,^119,124^ however, macrophages are also involved in the resolution of fibrosis via multiple mechanisms, including the clearing of excess collagen from damaged tissues and the secretion of collagenases that degrade ECM components.^81^

### Mast cells

Mast cells initiate and maintain inflammation.^111^ They may play an important part in the development of fibrosis^125^ and appear to be able to maintain a pro-fibrotic response, producing and storing many of the cytokines that promote fibrosis^14,111^ (see below under Cytokines), including TNF-α, IL-17 and TGF-β.^125,126^ Mast cells numbers are increased in fibrotic organs including the lung,^127^ heart and kidneys. Trautmann et al.^128^ demonstrated that mast cells stimulate fibroblast proliferation after attaching and directly releasing cytokines into their cytoplasm, suggesting an important mechanism by which fibrosis is promoted and maintained.

### T cells

The type of T cell response to inflammation controls the magnitude of fibrosis, with Th2 cells promoting the production of ECM and fibrosis, while Th1 cells are typically suppressive.^62,116^ Th17 cells are a subset of T reg cells that differentiate in the periphery in the presence of IL-1β, IL-6 and TGF-β.^129^ They secrete IL-17, a cytokine that is important for the activation and migration of immune cells, inducing them to secrete inflammatory cytokines and chemokines.^129^ Th17 cells are suppressed by the amino acid limitation response, which also enhances autophagy.^102^

### Inflammasomes

Inflammasomes are intracellular protein complexes that activate an inflammatory cascade by upregulating the production and maturation of inflammatory cytokines IL-1β and IL-18.^2,120,130^ Activated inflammasomes play a central role in fibrosis of organs including the liver,^131,132^ lungs^133^ and kidneys,^130^ upregulating α-SMA, connective tissue growth factor and collagen type I^131^. Inflammasomes serve as an inflammatory memory, however, it is not yet clear how they remain active in chronic fibrotic diseases.^2^

Inflammasomes are present in immune cells and a wide variety of cells in tissues, including myofibroblasts and fibroblasts, and are activated by an array of different signals from wounds and infection. Sterile activators include nuclear factor kB (NF-κB)^134^ and stimuli generated by cell death or damage, referred to as damage-associated molecular patterns (DAMPS), which signal the inflammasome via cell receptors. These diverse stimuli include ROS, adenosine triphosphate (ATP), mitochondrial DNA and proteins released from damaged ECM, such as hyaluronan, heparin sulphate and biglycan.^2,120^

Inflammasome activity is also regulated by secreted factors and by cell-to-cell interactions.^2^ In addition, some inflammatory cytokines that are released by dying cells, including TNF-α, IL-1α and IL-1β can act as DAMPS and activate inflammasomes.^2,120^ Intracellular proteins such as the chromatin associated protein high-mobility group box 1 (HMGB1) are also released by necrotic cells and act as DAMPS. Macrophages activated by TNF-α and TGF-β can also release HMGB1,^2^ activating inflammasomes and creating crosstalk between the production of inflammatory cytokines and the TGF-β signalling, with potential feedback loops and implications for fibrosis.

Inflammasomes directly and indirectly activate matrix production and fibrogenesis in tissue,^98^ and activate macrophages via production of IL-1β.^98^ It is of interest that IL-1β can stimulate NF-kB and p38 MAPK pathways and the resulting transcription of inflammatory cytokines including IL-6,^120,135^ perhaps leading to another feedback loop between inflammasome activation, IL-1β secretion and TGF-β production.

The inflammasome component nucleotide-binding domain and leucine-rich repeats containing pyrin domain 3 (NLRP3) is well studied. The NLRP3 inflammasome is a key player in sterile inflammation, and is associated with a range of auto-inflammatory and autoimmune diseases.^2^ Tissue damage and the accumulation of damaged mitochondria increases mitochondrial ROS production, which, along with other signals activates NLRP3 and stimulates processing of IL-1β pre-cursers into the biologically active form.^2,136,137^ NLRP3 also regulates ROS production by mitochondria.^138^ The activation of capase-1 by NLRP3 activates IL-1β and IL-18 precursors,^2,137^ and also causes the secretion of IL-1α and fibroblast growth factor 2^2^.

## Cytokines

Many cytokines have been associated with fibrosis, the most important being TGF-β. Other cytokines known to have involvement are TNF-α, IL-17, IL-1β and the anti-inflammatory IL-10.^139^ A combination of inflammatory cytokines upregulates expression of TGF-β receptors, and inflammation plays an important role in the development of fibrosis.^140^

### Transforming growth factor beta

Transforming growth factor beta (TGF-β) plays a central role in the pathology of arthrofibrosis^1^ and all fibrotic diseases,^141–144^ causing activation and proliferation of myofibroblasts, inhibition of collagen degradation, and an increase in ECM synthesis.^144,145^ TGF-β is produced by most cells, including inflammatory and effector cells^16,146^ and regulates immunity.^146^ It is secreted in a latent state, and must be activated by cleavage.^94^ Four isoforms are known and are involved in the regulation of cell proliferation, differentiation, adhesion, apoptosis, migration and fibrosis.^94,101,147^ TGF-β1 is the most abundant isoform, and is thought to be the most important in the pathology of fibrosis.^148^ Experimental induction of TGF-β causes excessive proliferation of fibroblasts in the knee joints of rats^1^ and stimulates the production of ECM, causing rat knee joints to become completely encased in fibrous tissue.^16^ ECM also stores latent TGF-β,^94^ which is released and activated by the stress between cell surfaces and ECM^149^ that occurs during the contraction of myofibroblasts.

Production of activated TGF-β is stimulated by oxidative stress,^138^ platelet degranulation^144^ and ROS released after injury or surgical insult.^94^ While ROS activates TGF-β and results in apoptosis,^150^ TGF-β also induces the production of ROS,^101,138,151^ thus creating a positive feedback cycle. This cycle may be exaggerated by another effect of TGF-β, the inhibition of the expression of antioxidant enzymes, including glutathione.^101^ The resulting higher levels of mitochondrial ROS significantly upregulates inflammatory cytokines and the production of inflammasomes.^138^ TGF-β also regulates T cell differentiation and proliferation and the activation and development of natural killer cells.^72^

Following the binding of TGF-β to its receptor complex, cytoplasmic signal transducer proteins called Smads are phosphorylated and promote the transcription of target genes in the nucleus.^138,144^ TGF-β also signals via non-Smad pathways including the extracellular signal-regulated kinase pathway (for review see ref. ^141^). In addition, TGF-β induces epigenetic modifications^147^ (see Epigenetic alterations below) and upregulates matricellular proteins, which interact with cell surface receptors and the ECM.^1^ However, the specific DNA sequences that are upregulated by TGF-β signalling is dependent on cell-specific DNA-binding co-factors.^94^

TGF-β drives a shift from Th-1 cells to pro-inflammatory Th-17 cells^72^ and upregulates the production of IL-11, a cytokine with a significant involvement in the development of fibrosis, in fibroblasts (see below). However, it can have different effects depending on the type of cells that secreted it, and the presence of other cytokines. For example, TGF-β secreted by regulatory T cells in the presence of IL-10 can inhibit inflammation and fibrosis,^62^ while TGF-β produced by macrophages is pro-fibrotic.^81^ Mitogen-activated protein kinases (MAPK) upregulate TGF-β expression in the presence of inflammatory cytokines,^147^ and form another feedback loop.

TGF-β is known to start a cascade of other downstream regulatory effects including a reduction in ECM degradation via the downregulation of a family of matrix metalloproteinases (MMPs),^152^ which include collagenases. Some MMPs are associated with the progression of fibrosis, however, some have a protective effect.^153,154^ TGF-β also induces tissue inhibitors of MMPs (TIMPS) that block ECM degradation and regulate MMP activity.^1,15,81^ MMPs play a key role in regulating a number of processes including ECM remodelling, proliferation, apoptosis and angiogenesis.^155^ MMPs are also induced by IL-17A, another cytokine with a significant involvement in the development of fibrosis^15^ (see below).

### Interleukin-1

IL-1β is believed to be an important mediator of fibrosis,^98^ influencing the migration of cells, adhesion, matrix metalloproteinase production and the expression of immune-modulatory genes.^156^ It is a powerful inflammatory cytokine that induces TGF-β^133,145^ and PDGF,^103^ driving the development of fibrosis^116^ following injury or infection. IL-1β is expressed in fibrotic tissues^53^ by a range of cell types, but is mainly produced by macrophages.^157^ However, it has been demonstrated that in fibrosis of the lungs IL-1β acts via TGF-β induction and signalling.^145^ In auto-inflammatory diseases IL-1β sets up a feedback loop such that it stimulates its own production.^158^

### Interleukin-6

IL-6 is a family of cytokines that have been associated with lung injury and the initiation of lung fibrosis,^159^ with fewer fibrotic changes seen in IL-6 deficient mice.^160,161^ Animal models show that this cytokine increases the expression of TGF-β receptors and their signal transduction,^162^ demonstrating another link between inflammation and fibrosis. IL-6 is essential for host defence against bacterial and viral infections, controlling T cell functions and survival. IL-6 also appears to be involved in the “memory” of inflammation^163^ and the development of chronic fibrosis.^161^

Recently, Schafer et al. demonstrated that IL-11 is strongly pro-fibrotic, driving the synthesis of the proteins involved in ECM production, contraction and other processes active in fibrosis.^143^ Production of IL-11 is upregulated by TGF-β. Neutralising antibodies to IL-11 and the deletion of IL-11 receptors inhibited the effects of TGF-β, suggesting new therapeutic targets for fibrosis.^143^ IL-11 is expressed by fibroblasts and other cells.^164^ It is a member of the IL-6 family of cytokines, and is also implicated in tumour progression.^165^

### Tumour necrosis factor alpha

TNF-α is thought to be important in the pathogenesis of fibrosis.^46,116,166–168^ It is a pleiotropic inflammatory cytokine^169^ that causes significant upregulation of TGF-β production^168,170^ and receptor expression^140^ and may stimulate fibroblast growth and collagen type I expression.^171^ TNF-α also causes fibroblast-like differentiation and inflammation,^138^ and PGE2 expression.^172^ TNF-α and IL-1 upregulate cyclooxygenase 2 (COX-2) synthesis in response to an insult.^158^ These cytokines also induce the expression of intracellular adhesion molecule-1 (ICAM-1),^158^ expressed in vascular endothelium, macrophages and lymphocytes, and associated with the development of fibrosis.^59^ Roberts et al.^152^ reported that TNF-α and IL-1β upregulated MMPs in vitro, potentially providing some anti-fibrotic effects, however, these cytokines also have pro-fibrotic effects. TNF-α may also be involved in the “memory” of insult, as TNF messenger RNA is able to remain elevated for more than 70 days.^173^

### Interleukin-17

IL-17 upregulates the production of TGF-β^174^ and inflammatory cytokines from chondrocytes and synovial fibroblasts,^175^ and promotes the survival of fibroblasts.^67^ IL-17 can directly induce the production of collagen type 1^139^ and disrupt ECM homoeostasis,^176^ while promoting MMP production.^176,177^ It is secreted by a number of cells types, primarily T-helper 17 (Th17), NK cells and mast and myeloid cells.^67,164^ The feedback loops between IL-17 and IL-6, TNF-α and IL-1 are considered important drivers of chronic inflammatory diseases,^139,175^ and suggest a mechanism for the development of chronic fibrosis. IL-17 acts as a pain sensitiser,^67^ induces monocyte migration and activates monocyte-derived macrophages to produce IL-1, TNF-α and PGE2.^178^

A number of other chemokines and cytokines including IL-13, IL-4 and IL-5 are associated with a higher risk of fibrosis, while IL-10 and IL-12 are protective.^62,116,161^ There is conflicting evidence for the role of interferon-γ.^161^

## Other pro and anti-fibrogenic mediators

NF-κB is a family of proteins that occur in the cytoplasm of cells in an inactive form. NF-κB regulates genes and cells involved in inflammatory responses,^179^ including the activation, differentiation and function of inflammatory T cells and inflammasomes.^120,134^ It directly and indirectly promotes Th17 differentiation, and dysregulated production of NF-κB is associated with a range of autoimmune and inflammatory diseases.^120^

NF-κB upregulates the transcription of chemokines and inflammatory cytokines including TNF-α, IL-1β and IL-6 in a range of innate immune cells, inducing inflammation.^120,157^ NF-κB in macrophages and fibroblasts is activated by TGF-β-activated kinase 1, as well as many of the inflammatory cytokines induced by it,^120^ leading to another feedback loop of inflammation and fibrosis. However, NF-κB is also necessary for inhibiting NLRP inflammasome activation in macrophages.^134^

### The 5′-adenosine monophosphate-activated protein kinase pathway

Adenosine monophosphate-activated protein kinase (AMPK) is a widely expressed member of the serine/threonine kinase family that is involved in energy regulation and the regulation of a range of genes involved in fibrosis.^180^ AMPK activation appears to regulate macrophages,^181^ limits ROS production,^182^ and is increasingly recognised as playing an important role in suppressing fibrosis.^180,181^ In addition, AMPK also appears to inhibit differentiation and proliferation of myofibroblasts and suppress collagen production.^180^ Stimulation of the AMPK pathway can occur via caloric restriction, exercise or medication.^181^

### Specialised pro-resolving lipid mediators

The discovery of resolvins, protectins, lipoxins and maresins has revolutionised the understanding of how inflammation is resolved. We now know that resolution is an active biochemical process mediated by these specialised pro-resolving LMs (SPMs), which act as a stop signal for inflammation and a return to homoeostasis.^109^ Specific SPMs have distinct anti-inflammatory, anti-microbial and pro-resolving effects.^183,184^ SPMs are derived from essential fatty acids, particularly omega 3 polyunsaturated fatty acids (ω-3 PUFA) found in fish oils and some plants, and are necessary in the human diet.^185^ SPMs have synergistic effects on immune function,^186^ downregulating the production of TNF-α and IL-1β,^184^ reducing pain, inhibiting neutrophil migration and protecting against uncontrolled inflammatory responses.^185,187^

Oral supplements of ω-3 PUFA result in biologically active levels of SPMs in serum including the important subtypes RVD1 and RVD2,^188^ and in synovial fluid, where SPM levels were negatively correlated with pain.^189^ These and other SPMs are able to switch macrophage phenotypes from pro-inflammatory to pro-resolving (ref.^183^ and references within), and reduce the expression of inflammasomes.^184^ SPM profiles in patients correlate with outcomes, with a lack of them linked to delayed resolution of inflammation.^184^

Importantly, SPMs were shown to be anti-fibrotic in organs including the kidney^190^ and liver.^191^ PDGF-induced myofibroblast proliferation is inhibited,^190^ along with the production of inflammatory cytokines, and SPMs may represent an important new treatment for fibrosis.^190^ Although SPMs have a short half-life in vivo, more stable synthetic analogues have been developed,^192^ and may become a useful therapy for a range of inflammatory diseases and fibrosis.

### Nonsteroidal anti-inflammatory drugs

Nonsteroidal anti-inflammatory drugs (NSAIDS) may prolong chronic inflammation if used for more than 48 h because the resulting inhibition of COX-2^193^ causes inhibition of resolvin production and other SPMs.^56,194^ COX-2 is an important anti-fibrotic enzyme.^195^ The chronic inflammation induced by long-term NSAIDS use is known to activate fibrosis of the kidneys^16,196^ and lung.^195^ However, it is not known how NSAIDS use affects arthrofibrosis, despite it being a commonly prescribed treatment.^197^ Importantly, aspirin is an exception as it acetylates COX-2, favouring the production of lipid mediator precursors over pro-inflammatory prostanoids.^192^

### Hypoxia

Hypoxia is the lack of sufficient oxygen to carry out normal cellular processes, and occurs in tissue surrounding wounds.^198^ Hypoxia is believed to be important in the development of fibrosis,^198,199^ via wide-ranging effects. It promotes SMAD 2 phosphorylation and expression of α-SMA, collagen type 1, MMP-2 and TIMP-1.^200^ The lower pH created by increased levels of lactic acid may be important in activating TGF-β and myofibroblasts.^201^ Furthermore, fibrotic tissue has reduced vascularity, resulting in permanently hypoxic tissues and another positive feedback cycle where lactic acid and fibrotic mediators are continuously expressed.^198,201^

Many of the effects of hypoxia are driven by hypoxia-inducible factor-1 (HIF-1), a protein that is a key regulator of genes in hypoxic tissue.^202,203^ It is upregulated and stabilised in response to ROS^179^ and in tissue with low oxygen levels^200^ and is important in both normal wound healing and in fibrosis.^198^ HIF-1 increases SMAD3 signalling and thereby TGF-β signalling,^204^ and upregulates connective tissue growth factor^198^ and genes involved in ECM deposition.^205^ Inhibiting HIF-1 inhibits myofibroblast differentiation^201^ and reduces transcription of collagen type 1.^203,206^ HIF-1 is known to be upregulated in cardiac fibrosis^202^ and contributes to the progression of liver disease to liver fibrosis.^207^

### Reactive oxygen species

TGF-β, IL-1 and TNF-α stimulate ROS production from a range of cell types including fibroblasts, and TGF-β can also suppress the production of antioxidant enzymes.^208^ TGF-β promotes ROS production^209,210^ and in a feedback effect, high levels of ROS stimulates TGF-β production^94^ and causes more damage, cell death^198^ and the release of cell fragments that act as DAMPs, causing activation of NF-κB and increased expression of inflammatory cytokines. ROS can also directly and indirectly activate MMPs.

### Proteases

MMPs and TIMPS have an important role in fibrosis by controlling matrix degradation.^104,117^ They are produced by macrophages and can have pro- or anti-fibrotic properties depending on the microenvironment and cytokine expression.^116,117^ MMP1, MMP8 and MMP13 appear to be important in the context of fibrosis due to their ability to cleave collagens 1, 11 and 111.^104^ Mature ECM with extensive cross-linking is resistant to degradation, and appears to promote the survival of myofibroblasts and further collagen deposition.^117^ Fibrotic tissue also has reduced vascularisation, and cells within fibrotic tissue express a hypoxia-specific gene and proteins that indicate oxidative stress (see above).^13,101^

### Substance P

Substance P is an immunomodulatory neuropeptide released by a variety of cells immediately following injury. Substance P and its receptor neurokinin-1 increase pain transmission, and their synthesis is upregulated in response to TNF-α and IL-6.^211^ In a positive-feedback loop substance P stimulates mast cells,^111,212^ upregulates mediators of inflammation, cell proliferation^211^ and antiapoptosis,^213^ and many pro-fibrosis genes.^214^ Substance P also increases the expression of collagen type 1 and α-SMA,^215^ upregulates TNF-α and promotes adhesion of cells.^216^ A high ratio of sensory nerves expressing substance P compared to sympathetic nerves was found in tissue from arthrofibrotic knees, suggesting a major role for this peptide.^57^

## Epigenetic alterations

DNA methylation and histone modifications alter access to DNA, thereby significantly changing the rates of gene transicription.^147^ These epigenetic changes, together with the upregulation of micro-RNAs^147^ and other noncoding RNAs, are significant in many diseases^179,217^ including fibrosis.^104,108,118,218–220^ They typically occur in response to environment changes including an increase in ROS,^179^ resulting in dysregulated cell signalling pathways^221^ that can affect collagen expression,^104^ apoptosis, the immune system and other fibrotic pathways.^218,220^

Epigenetic modifications are stable and passed on to subsequent generations of cells^217,222^ unless reversed by specific agents.^223^ DNA methylation enzymes add methyl groups to cytosine bases, blocking gene transcription, and TGF-β drives increased methylation of anti-fibrotic genes and decreased methylation of fibrotic genes.^147^ Increased DNA methylation is associated with fibrosis of the heart,^220^ lungs^195^ and other organs.^147^ It triggers myofibroblast activation and resistance to apoptosis,^222,223^ and can also trigger histone acetylation, strengthening pro-fibrotic effects.^222^

However, the effects of DNA methylation are sometimes indirect. For example, Evans et al.^195^ demonstrated that the hypermethylation of a COX-2 transcriptional regulator in lung fibrosis resulted in suppressed COX-2 expression and a fibrotic phenotype. In addition, hypermethylation of micro-RNA promoter regions can result in the upregulation of genes normally supressed by micro-RNA, causing fibrosis.^223^

Demethylation is triggered by translocation enzymes, and these are downregulated in liver fibrosis, suggesting that an imbalance between methylation and demethylation enzymes contributes to fibrosis.^219^ In a similar fashion, sirtuins are natural enzymes that remove acetyl groups on histones, providing protection from a range of diseases including fibrosis.^224^

Histone modifications include both acetylation and methylation of nuclear histones that package DNA, with the former promoting gene transcription^218^ and the latter typically suppressing it.^147,220^ TGF-β is known to alter histone modifications, and acetylation of histones is associated with myofibroblast activation, increased production of inflammatory cytokines^220^ and increased SMAD3 transcription.^225^ Non-histone protein methylation can also alter the activity of transcription factors and promote TGF-β signalling by decreasing Smad7 protein stability.^226^ Smad7 is an inhibitor of TGF-β expression.

TGF-β also upregulates a wide range of pro-fibrotic micro-RNAs and long noncoding RNAs, and downregulates anti-fibrotic micro-RNAs.^147^ In liver disease long noncoding RNAs and other noncoding RNAs can promote or reverse fibrosis via a variety of mechanisms, including upregulation of CTGF^227^ and TGF-β signalling.^228^

Epigenetic alterations are likely to be significant factors in persistent active arthrofibrosis, as has recently been shown for lung fibrosis,^195^ other fibrotic diseases^108,147^ and cardiorespiratory abnormalities from hypoxia-induced DNA methylation and persistent increases in ROS.^229^

## Histopathology

Histochemical and immunohistochemical studies have significantly advanced the understanding of the pathogenesis of arthrofibrosis, and fibrosis in general, demonstrating alterations in tissue composition and structure and cell activity. Commonly used histological stains are easily applied and readily visualise fibrotic tissue and ECM,^218^ permitting patient diagnosis and visualisation of treatment efficacy in animal models. Although the results from arthrofibrosis studies have been variable,^19^ possibly due to differences in the type of biopsy tissue and the location and extent of fibrosis of donor patients, these studies have nonetheless provided important information.

Early arthrofibrosis research found increased collagen accumulation in the IFP,^230,231^ with later studies reporting high numbers of myofibroblasts positive for the presence of α-SMA^232–234^ and a proliferation of fibrotic connective tissue.^234^ Later, Freeman et al.^13^ found that fibrotic tissue from the knees of arthrofibrosis patients contained heterotrophic ossification, limited vascularity and increased numbers of mast cells expressing fibroblast growth factor.

The number of myofibroblasts in tissue from arthrofibrotic knees can be ten times higher than in healthy subjects.^233^ Ruppert et al.^234^ observed co-localisation of β-catenin and the tight junction protein ZO-1 in myofibroblasts which may cause increased adhesions and mechanical loading of cells. This finding can be applied to distinguish arthrofibrosis from other conditions when tissue samples are available, with a threshold of 20 myofibroblasts expressing β-catenin per high powered field of view.^234^

Other histopathology studies suggest additional pathways involved in the pathogenesis of arthrofibrosis. Faust et al.^232^ found increased expression of xylosyltransferase-I mRNA in the synovial membrane of arthrofibrotic knees treated with TGF-β1, along with increased α-SMA and collagen. Xylosyltransferases catalyse the production of proteoglycans associated with fibrosis, and are involved in tissue remodelling and myofibroblast proliferation.^232^

Koeck et al.^57^ reported an increased ratio of sensory nerves to sympathetic nerves in tissue from the anterior of arthrofibrotic knees compared to OA knees. Antibodies to substance P were used to indicate the presence of sensory nerves, suggesting that hyperinnervation and high levels of substance P may be significant contributors to active arthrofibrosis.^57^

## Current treatments and new therapeutic outlooks

### Non-pharmacological treatments

Arthrofibrosis research has often focused on treatments that address the structural pathology of the condition. These treatments include surgical interventions, such as arthroscopic lysis and debridement of ECM, open surgery to remove ECM and release of tendons and ligaments, and MUA.^35^ Other treatments include bracing, corticosteroids and physical therapy^3,33^ (Table 2).

**Table 2:** List of existing and potential new therapies for treating arthrofibrosis, with a summary of the associated benefits and risks

Therapies	Benefits/risks
*Dietary approaches*	
Omega 3 fatty acids in fish or supplements	Necessary for the production of SPMs vital for resolution of inflammation. Thins the blood, but typically no risks are associated within recommended daily limits.
Capsaicin (in peppers) and sulphoraphane (in cruciferous vegetables)	May reverse differentiation of myofibroblasts, sulphoraphane may prevent fibroblast differentiation. No risks are associated within recommended daily limits.
Resistant fibre	Gut bacteria produce short-chain fatty acids which counter inflammation. No risks are associated within recommended daily limits.
Low-sugar intake	Reduces inflammation. Typically no associated risks.
Soy products	Contains anti-inflammatory compounds. Reduced levels of TGF-β and lung fibrosis in rats. Benefits not established for treating fibrosis. Typically no risks are associated within recommended daily limits.
Potassium	May help prevent fibrosis, negative correlation between high levels of serum K^+^ and liver fibrosis. Typically no risks are associated within recommended daily limits.
Intermittent fasting	Protective against fibrosis of organs, suppresses inflammation, IL-1, IL-6 and TNF-α and inflammasomes. Typically no risks are associated. May be difficult to follow.
*Pharmaceuticals*	
Oral and injected corticosteroids	Downregulates inflammation and possibly TGF-β. Increased risk of infections, suppressed adrenal gland hormone production, can cause high-blood pressure and liver damage etc if long-term.
TGF-β antibodies?	Several TGF-β neutralising antibodies and receptor blocking antibodies are in clinical trials. May prove to be effective therapies for arthrofibrosis.
IL-1 antibodies and IL-1 receptor antagonists	Have been successfully used to prevent post-operative arthrofibrosis in small studies. Shown effective at reducing lung fibrosis in animals (Gasse et al. 2007). Efficacy in the treatment of existing arthrofibrosis not known.
Halofuginone?	Inhibits Smad3 signalling by TGF-β. Suppresses collagen type I, fibroblasts and Th17 cells. Causes GI bleeding, enteric coated capsules recommended. Benefits and risks not established for treating fibrosis.
Low dose aspirin?	Induces production of SMPs. Can cause GI symptoms in some, enteric coated capsules recommended. Blood thinner.
TNF-α antibodies?	Reduces pain, inflammation, fibrosis and serum TGF-β in animals. Increased risk of infections. Benefits and risks not established for treating fibrosis.
Pirfenidone	Therapy for lung fibrosis, anti-fibrotic and anti-inflammatory, downregulates fibroblasts, collagen, alpha smooth muscle cell actin. Diarrhoea, photosensitivity, GI symptoms and liver toxicity in some.
Nintedanib	Therapy for lung fibrosis, anti-fibrotic, downregulates collagen. Diarrhoea, GI symptoms and liver toxicity in some.
Ketotifen?	Used to treat asthma, modifies mast cell activity. Results of small trial for elbow arthrofibrosis shows no effect.
Metformin?	Used to treat type II diabetes. Reduces TGF-β production, interferes with TGF-β signalling, reduces collagen deposition and proliferation of fibroblasts. Reduces fibrosis of organs.
Collagenase	May damage articular cartilage, ligaments and tendons, but trials show no negative effect on these structures. Repeated injections needed, increases ROM in shoulder arthrofibrosis. More trials are needed.
Substance P antagonists?	Used to alleviate nausea. In animal studies downregulates pro-fibrotic genes in joints and reduces fibrosis and inflammation of the colon.
Interferon β therapy?	Downregulates NLRP3 inflammasomes. Benefits and risks not established for treating fibrosis.
Epigenetic drugs?	May reverse myofibroblast differentiation and DNA and histone modifications that cause persistent fibrosis. Benefits and risks not established for treating fibrosis.
*Surgical approaches*	
Arthroscopic lysis and debridement of ECM	Removal of adhesions and ECM can increase long-term ROM. Risk of adverse outcomes from the inflammatory response and worsening fibrosis. Infection, blood clots. No method to determine how individual patients will respond.
Manipulation under anaesthesia	Disruption of adhesions can increase long-term ROM. Risk of adverse outcomes from the inflammatory response and worse fibrosis. Risks include heterotrophic ossification, bone fracture, damage to prosthesis, ligament rupture and blood clots.
Open surgery	Removal of adhesions and ECM can increase long-term ROM. Risk of adverse outcomes from the inflammatory response and worse fibrosis. No method to determine how individual patients will respond.
*Physical therapies*	
Bracing	May be needed for healing. Risk of adhesions forming due to lack of movement.
Exercise, physical rehabilitation therapy	Increases strength and ROM. Intensity should be adapted according to resulting inflammation in individuals. Risk of increasing inflammation and fibrosis when limits are exceeded.
Continuous passive motion	Remains controversial. May help to avoid MUA, likely more beneficial for patients with arthrofibrosis than for those without. Must be well controlled to prevent damage to tendons and ligaments from forced over-bending.
*Other*	
Mesenchymal stem cells?	Modulate the immune system, inhibit the production of inflammatory cytokines. Age and origin may affect the outcome. May differentiate into fibroblasts. Can encourage tumours. Benefits and risks not well established for treating fibrosis.

For other potential therapies, including those that inhibit TGF-β signalling, see main text. Patients should always receive medical advice before adopting new treatments or diets and before altering treatment as this may alter current treatments or comorbidities. Some of these approaches are not well established for treating fibrosis, but are known to reduce inflammation. *SPMs* special pro-resolving lipid mediators, *RDI* recommended daily intake, *ROM* range of motion, *?* a pharmaceutical therapy that is currently used to other conditions, which has potential for treating arthrofibrosis

#### Continuous passive motion

Post-operative use of continuous passive motion (CPM) is sometimes prescribed to increase ROM,^52,235^ but remains controversial, most likely due to the associated expense and inconvenience.^236,237^ This results in many patients not having access to regular CPM.^235^

Ferretti et al.^238^ used antibody-induced arthritis in rabbits to show that CPM lowers levels of inflammatory IL-1β, increases anti-inflammatory IL-10 and decreases MMP-1 compared to immobilisation. This suggests that the mechanical forces created by CPM reduce inflammation and pain, and may reduce damage to cartilage. In addition to potentially increasing ROM, CPM may further assist post-operative patients by lowering the risk of arthrofibrosis via these effects, particularly in patients that are not fully mobile.

Unfortunately, the efficacy of CPM is difficult to determine as trials differ in their duration, timing and length of CPM treatment. The number of participants in CPM trials is particularly important because only a small proportion of patients develop post-operative arthrofibrosis, and it is only in these patients that a significant gain in ROM would be anticipated. A 2014 Cochrane Review that analysed 24 randomised controlled trials of CPM following TKR over 1–17 days found that CPM use may slightly increase ROM and quality of life, although these were not clinically relevant, and low-quality evidence to indicate that CPM reduces the risk of MUA by 4%.^236^

This review did not exclude trials based on the quality of the research, and only ten studies blinded assessors to CPM use. Increased ROM is the primary reason for CPM treatment, and of the ten trials that reported short term (0–6 weeks) effects on ROM, only five used a blinded assessor.^236^ Even fewer studies used a blinded assessor for the reported medium term (6 weeks to 6 months) and long-term (over 6 months) ROM. The lack of well controlled studies makes it difficult to draw conclusions.

In addition, Chaudry et al.^237^ observed that the 2014 Cochrane Review results may not apply to patients with “unique considerations”, particularly those that have undergone an MUA and for whom there is a higher than usual risk of adhesions. It is also likely that for patients that develop active arthrofibrosis, more than 1 or 2 weeks of frequent CPM are required to counter the continuing formation of ECM and adhesions. Quality research into the efficacy of CPM for patients with arthrofibrosis, rather than the general orthopaedic patient community, is urgently needed.

#### Surgery and MUA

Arthroscopic lysis of ECM is the most commonly performed treatment for arthrofibrosis,^33,35^ and MUAs are also frequently performed either on their own, or during arthroscopic surgery. These treatments can be successful, perhaps not only because of the obvious benefit of removing the physical restriction to ROM. The release of stress created by the lysis of ECM can potentially interrupt the feedback loop between myofibroblast activation due to mechanical loading and resulting contraction, in the process encouraging apoptosis of myofibroblasts.^113^ In addition, the removal of ECM during surgery removes bound pro-fibrotic mediators, including TGF-β.^108^

Nonetheless, the benefits of surgical lysis and MUA should be tempered by an understanding of the problems associated with these procedures. Both treatments damage tissues, and tissue injury stimulates an inflammatory response^239^ that may cause further fibrogenesis. One study found that patients with elbow injuries had significantly less ROM if they were treated surgically instead of non-operatively at 12 weeks.^240^ Some authors state that MUAs should not be performed due to the risk of fractures, rupture of tendons and cartage damage,^35^ while others warn that delayed MUA or manipulation that is too aggressive can lead to these complications as well as ossification of the medial collateral ligament and quadriceps.^6^

Daluga et al.^42^ found that MUAs significantly increased heterotrophic ossification in an MUA group compared to a control group based on radiographic observations. This is probably due to tearing of tissues during the process and bleeding. One review found that MUAs had caused hemarthroses, wound dehiscence, subdural haematoma, bone fracture and two fatal pulmonary emboli, but reported that most studies did not include enough patients to show up these risk factors.^36^

It is difficult to determine how successful surgery is for treating arthrofibrosis.^6^ Patients differ widely in the severity of symptoms, degree of inflammation and extent of fibrosis within and around the joint, and surgical treatments also vary greatly in extent. Measures of outcomes and classification of patients also differ,^6^ affecting reporting.

In addition, studies of surgical procedures to treat arthrofibrosis are often poor quality.^36^ They frequently have small sample sizes, sampling bias and reporting bias,^5^ and are typically not comparative^33^ or controlled, complicating the interpretation of these procedures.^36^ The reported high rates of success of surgeries to treat arthrofibrosis and lack of adverse outcomes do not correlate well with outcomes reported by patients on patient websites such as KNEEguru^241^ and indeed it is understood that published results of knee surgery including TKRs by specialised surgical centres may be misleading and overly optimistic.^68,242^

It is known that surgery sometimes worsens symptoms of arthrofibrosis,^23^ but these cases may not be reported. Some patients are removed from studies of surgical outcomes after a diagnosis of “complex regional pain syndrome”, a condition with no established diagnostic criteria (see above). Regardless of diagnosis, these patients should be included when reporting the results of surgery, both initial surgery to treat an injury, and surgery to treat arthrofibrosis. Not doing so is misleading and makes interpretation of results difficult.

Studies often do not specify how ROM is evaluated, and changes in ROM are sometimes reported as relative measures.^33^ Additionally, it is often not reported how severe ROM limitations are before treatment.^33^ ROM results are often averaged, obscuring any patterns that may exist in treatment outcomes. For example, patients with more severe ROM limitations may not benefit as much as those with relatively good ROM before treatment, but this type of outcome typically can not be determined from the published data.

A recent review of the literature on treatments for arthrofibrosis included 647 patients in 25 studies, however, only 241 patients (37%) had their ROM established using a goniometer.^33^ More than half of patients were successfully treated without surgical intervention, and of those that had arthroscopic lysis of ECM 6% required more than one procedure for ROM deficits.^33^ Of the 25 studies examined in this review only 6 reported statistically significant improvement in ROM following treatment.

Everyone is affected by bias, and although well-intentioned, surgeons have a vested interest in reporting positive outcomes from their treatments. It is, therefore, important that studies are well designed to control for bias.^243^ Unfortunately, double blind trails are difficult where surgery is involved, but sham surgeries have been successfully performed to demonstrate the lack of effectiveness of chondroplasty compared to placebo for the treatment of OA.^244,245^ Chondroplasty is débridement (shaving the cartilage), typically with lavage (wash-out) of the joint, and remains the most widely performed knee surgery for arthritis, despite randomised trials showing no difference in pain or functional status over non-surgical controls.^243,246^

An additional complicating factor is a history of multiple injuries or surgeries to a particular joint, which appears to increase the risk of a patient developing post-operative arthrofibrosis. Unfortunately, the effect of previous surgeries on the outcome of surgery or MUA is seldom mentioned in published studies, and is rarely the main focus of a study.^6,11^ However, Ipach et al. (2011) showed significantly worse outcomes from an MUA for patients that had previously undergone one or two surgical procedures. Sachs et al. stated that 18% of patients that had multiple surgeries developed arthrofibrosis, compared with 5% that had one surgery to repair an ACL.^22^

In a review of patients undergoing treatment for arthrofibrosis, Werner et al.^5^ showed that each additional procedure performed during the surgery incrementally increased the rates of arthrofibrosis. While complications from surgical treatment of arthrofibrosis are often not reported, or are poorly reported,^36^ some authors suggest that the return of arthrofibrosis is common following these procedures.^32^

For example, a young female patient with minimal loss of ROM but considerable pain underwent three surgeries to lyse ECM and adhesions, however, each surgery appeared to worsen the pain and inflammation despite a focus on decreasing inflammation. There were serious complications after the final surgery including poor healing, neuropathy, quadriceps atrophy, unresponsive swelling and excessive pain.^12^ This patient demonstrates the important point that systems to diagnose and grade the severity of arthrofibrosis based on ROM will fail to include some patients that have severe pain and disability from the condition, but only limited ROM loss. In this case the patient’s flexion only dropped below 100° for a 1-week period after the third operation, and her extension was never worse than 0°.

Nonetheless, surgical removal of ECM from the joint may assist when the inflammatory response that arises from surgery can be controlled. ECM promotes survival of myofibroblasts and the deposition of collagen, and once mature is resistant to degradation.^117^ This likely explains why some patients that have surgical lysis and removal of ECM recover. However, patients with minimal loss of flexion may be best treated with conservative, non-surgical interventions given a basal risk of complications of around 4.7% from arthroscopic knee surgery,^247^ together with the risk of recurring arthrofibrosis. The overall risk of surgical complications such as infection is significantly higher for young men than for women over 40.^247^

The inflammatory reaction to surgery and foreign material in implants could potentially be controlled by the use of implant coatings that interfere with macrophages.^84^ In a similar manner, anti-fibrotic drugs could also be developed as coatings for implants to prevent post-operative arthrofibrosis.^83^ Halofuginone is a promising anti-fibrotic candidate (see below) and implant coatings of halofuginone have been shown to reduce the fibrosis in rats.^248^

Alternatively, slow release capsules of anti-fibrotic drugs or scaffolds containing drugs could be introduced at the time of surgery, or after arthrofibrosis develops. Arsoy et al.^249^ successfully used surgically implanted intra-articular hydrogel scaffolds containing rosiglitazone in a rabbit model of arthrofibrosis to reduce loss of ROM from trauma. This approach could be used to prevent the return of arthrofibrosis at the time of surgery to lyse adhesions and remove ECM. Some of these approaches may increase the risk of infection, and additional anti-microbial coatings or implants containing antibiotics^84^ could be applied.

## Pharmacological treatments

Because fibrosis is caused by an imbalance in cytokine production activated by high levels of TNF-α, IL-1,^139^ TGF-β and other mediators an effective therapeutic approach may involve regulating cytokines and mediators to favour resolution. However, despite the understanding of the pathogenesis of fibrosis, there are no effective therapies to halt fibrosis, and none to cure it.^16,107^ This situation may soon change, with a range of pharmacological therapies in clinical trials for the treatment of fibrosis of organs.^17^

The large number of potential therapeutic targets^116^ may have complicated and slowed progress. A review of the mechanisms and experimental approaches to kidney fibrosis in diabetic patients found 17 mechanisms and 80 experimental approaches to inhibit ECM formation.^250^ It is possible that multiple pathways need to be targeted together for the most effective outcome,^17,251^ particularly when fibrosis is well established. Nonetheless, it is also possible that targeting one or two key mediators early in the process may halt the dysregulation that leads to permanent active fibrosis, and targeting epigenetic modifications could potentially turn active arthrofibrosis into residual arthrofibrosis. Arthrofibrosis may be a useful candidate for testing new therapies since it can be identified early after surgery, unlike fibrosis of organs that are typically detected late in the disease process.

Although fibrosis has been considered an irreversible condition, it is significant that some studies show that fibrosis can be resolved and sometimes reversed at least partially in animal models and humans, demonstrating that the synthesis and degradation of ECM is dynamic and can proceed in both directions.^45,107,108,117,252^ For example, in the knee, linear scarring sometimes occurs along the arthroscopic portal paths. This peaks at 6 months, but after a year is no longer present in half of patients,^253^ suggesting that fibrosis is a frequent reaction to surgery that often resolves without treatment.

Treatment of lung fibrosis has improved recently with the introduction of pirfenidone and nintedanib.^251^ Pirfenidone downregulates inflammatory cytokines including TNF-α, IL-1β and IL-6 in addition to its anti-fibrotic effects of blocking TGF-β stimulated collagen production, production of PDGF, α-SMA and fibroblast proliferation.^251^ These treatments may be beneficial for treating arthrofibrosis either on their own, or in combination with other therapies, since therapies for treating organ fibrosis are likely to be effective for the treatment and prevention of arthrofibrosis.

The role of hypoxia in the development of arthrofibrosis requires research. Future treatments for fibrosis may target hypoxia-inducible factor-1 or its downstream signalling^202^ to prevent areas of reduced vascularity and positive feedback with TGF-β production and myofibroblast activation. Another target for early intervention is substance P. Recent research has found that knockout of the receptor for substance P attenuates liver fibrosis in animal models,^213,254^ while other animal studies found that a receptor antagonist for substance P downregulated some pro-fibrotic genes in joints^214^ and reduced fibrosis and inflammation of the colon.^255^ Substance P antagonists are routinely used to alleviate nausea.^214^

### Anti-inflammatories

Although anti-inflammatory medications do not halt fibrosis of organs,^107^ they are nonetheless the only currently prescribed medications for treating arthrofibrosis. Aspirin has been shown to inhibit production of NF-κB via the IKK receptors,^120^ and importantly, aspirin triggers the production of more stable and potent SPMs.^256–258^ In animal models of liver fibrosis aspirin reduced levels of pro-fibrogenic mediators and the progression of fibrosis.^259^ As mentioned previously, NSAIDS other than aspirin have been shown to disrupt class switching of COX-2, preventing the production of SPMs and inducing long-term inflammation.^56,256,257^

Corticosteroids, particularly glucocorticoids, are frequently prescribed to patients with arthrofibrosis in oral or injected form, and reduce symptoms. Glucocorticoids such as glucocorticoid dexamethasone and annexin peptides and their derivatives downregulated inflammation in lung fibrosis and reduced the infiltration of neutrophils and monocytes.^260^ The peptide Ac2-26, an annexin derivative, inhibited collagen deposition as well as TGF-β and TNF-α in mouse models.^260^ Glucocorticoids also inhibit the DNA-binding of NF-κB.^120^ Prednisolone reduces the expression of adhesion molecules, limits tissue damage and may downregulate TGF-β in liver fibrosis, particularly when used together with azathioprine.^61^

IFN β therapy appears to be an effective treatment to downregulate NLRP3 inflammasomes.^261^ Several other treatments that target the NLRP3 inflammasome, some of which are currently available, are reviewed by Shao et al.,^261^ and may be a useful anti-inflammatories for treating or preventing arthrofibrosis in high-risk patients.

Another medication that is currently available may also assist in treating arthrofibrosis. Ketotifen is an antihistamine used to treat asthma, and modifies mast cell activity. Monument et al.^262^ found that ketotifen treatment reduced arthrofibrosis in rabbits by decreasing the numbers of mast cells and myofibroblasts. However, a recent clinical trial to evaluate the use of ketotifen to reduce elbow contracture after injury demonstrated no significant increase in ROM at 12 weeks in a group treated with oral ketotifen compared to the control group.^240^ This result is difficult to interpret due the low numbers of patients, with only 34% of the ketotifen treatment group having surgery. The number of patients that developed arthrofibrosis was not reported. More trials will be necessary to determine if ketotifen can prevent arthrofibrosis, and evidence suggests that it should be administered soon after surgery or injury for the most effective outcome.^14^

### Modifiers of TGF-β signalling

Although TGF-β is the primary inducer of fibrosis,^16^ blocking its production is complicated by the many essential biological roles it plays.^94,144^ Some studies therefore aim to modify downstream signalling to minimise side-effects. However, there are many different aspects of TGF-β production, activation and signalling that can be targeted therapeutically, with antibodies, antisense oligonucleotides, ligand competitive peptides and inhibitors in clinical trials.^94^

Some medications already in use for other conditions may have therapeutic potential for arthrofibrosis. Metformin has been used extensively to treat type II diabetes and has risen to prominence after it was found to reduce death from all causes.^263^ Metformin appears to reduce TGF-β production^159,264–266^ and interferes with TGF-β signalling,^264–266^ reducing fibrosis of the kidney,^265,267^ lung,^159,264^ heart^263,266^ and liver.^268,269^ Zheng et al.^270^ found that metformin reduced fibrosis of tendons in rats, which had reduced adhesions and α-SMA expression in tendons compared to controls. Furthermore, metformin did not inhibit healing. In vitro analyses in the same study indicated that metformin decreased levels of Smad 2/3 phosphorylation and extracellular signal-regulated kinase 1/2, suggesting that metformin targets canonical and non-canonical pathways in TGF-β signalling.

In vitro and animal studies show that metformin reduces collagen deposition and proliferation of fibroblasts after initiation of fibrosis compared to non-treated controls,^159,264,270^ with some of these studies also showing reduced levels of α-SMA expression. Metformin reduced levels of inflammatory cytokines, including IL-6, IL-17 and IL-18^263^ as well as TNF-α in animal models of lung fibrosis.^264^ Qin et al.^271^ reported that metformin decreased levels of messenger RNA for inflammatory cytokines in vitro and reduced alkaline phosphatase activity, a marker of osteogenesis, in human ligament fibroblasts.

Metformin has also been shown to suppress expression of hypoxia-inducible factor-1 and to activate the adiponectin-5′-AMP-activated protein kinase (AMPK) pathway.^272^ Together these results indicate that metformin may have significant therapeutic potential for the treatment of arthrofibrosis, and the well-known safety profile of this medication makes it particularly attractive.

Halofuginone also suppresses TGF-β, but does not have the well-known safety profile of metformin. Halofuginone directly inhibits Smad3 signalling by TGF-β.^15,114^ Smad3 upregulates the production of pro-fibrotic proteins and miRNAs,^147^ and is considered essential in the fibrotic process.^144^ Halofuginone reduces collagen type I, suppresses myofibroblast proliferation and has been shown to resolve and reverse established fibrosis in animals models.^15,273^ The reduction in collagen type I synthesis appears to be the result of inhibited gene expression^15,274^ and only occurs in soft tissues, not in bone.^15^

In addition, halofuginone inhibits the development of Th17 cells,^44^ decreasing Th17 cell numbers and inflammatory cytokines via AMP-activated protein kinase-mediated NF-κB p65 inactivation.^275^ Halofuginone has undergone clinical trials to treat Duchenne muscular dystrophy and several forms of cancer using an oral encapsulated form to prevent gastric bleeding. Injections of halofuginone are also effective in animal models, and could potentially be used post-surgically in the joints of patients at risk of developing arthrofibrosis. The ability of halofuginone to supress the production of TGF-β by fibroblasts^15^ may be particularly important for treating active arthrofibrosis, and its ability to trigger the dissolution of collagen and decrease established fibrotic conditions^15^ could potentially assist patients with well-established arthrofibrosis.

Another modifier of the expression of activated TGF-β was recently demonstrated to have some efficacy in rat models of kidney fibrosis. MK-0429 is thought to downregulate some or all of the TGF-β cell receptors necessary for activation of TGF-β, leading to reduced collagen type 1 production.^276^ This compound is taken orally, and was first developed to treat osteoporosis.

Other therapies that inhibit TGF-β signalling are discussed in Lichtman et al.,^148^ Lee et al.^277^ and Xu et al.^94^

### Epigenetic regulators

Drugs that target epigenetic modifications hold significant promise for treating and even reversing fibrotic conditions due to the ability to alter gene transcription in many pathways simultaneously.^278^ This potential has recently been recognised, and epigenetic drugs are beginning to be tested for efficacy in a range of fibrotic diseases. For example, Evans et al.^195^ showed that inhibition of DNA methylation enzymes could reverse the downregulation of COX-2 expression in lung fibroblasts and de-activate them.

Myofibroblast differentiation is a particularly attractive target,^46^ and epigenetic reprogramming and de-activation of myofibroblasts could control dysregulated TGF-β signalling, inflammatory cytokine production and ECM synthesis and cross-linking.^219^ However, further research is needed to clarify the functions of specific inhibitors and promoters, as they can affect many cell types and can have off-target effects including the deacetylation of proteins.^220,279^ Zeybel et al.^278^ demonstrated that myofibroblasts in liver fibrosis could be targeted using liposomes coated with myofibroblast-specific antibodies to deliver epigenetic drugs, potentially side-stepping potential issues with off-target effects.

Histone deacetylase inhibitors have been effective in treating liver and kidney fibrosis in rodents,^218^ and Schuetze et al.^279^ demonstrated that diverse histone deacetylase inhibitors were able to suppress proliferation of cardiac fibroblasts in vitro. In addition, the knockdown of a noncoding RNA was shown to reduce liver fibrosis in mice by reducing TGF-β signalling^228^ and sirtuins were shown to downregulate inflammatory cytokines and M1 macrophages via deacetylation of a NF-κB subunit,^220^ ^refs therein^. Zhang et al.^224^ demonstrated that abnormally high levels of sirtuin 6 suppressed myofibroblast differentiation in human cells in vitro by inhibiting TGF-β and NF-κB signalling pathways.

Currently prescribed medications with a known safety profile can be readily trialled as epigenetic regulators. Valproic acid is currently prescribed for migraines and other conditions, and is a histone deacetylase inhibitor.^218^ Li et al.^227^ showed that valproic acid could reverse human liver myofibroblast activation in vitro, with the possible involvement of noncoding RNAs. Long-term valproic acid treatment also reduced ROS, TNF-α, IL-6, IL-1β and NF-κB activity and expression in diseased rat hearts.^280^ Other epigenetic compounds are reviewed by Nebbioso et al.^217^ and van Beneden et al.^218^

Dietary phytochemicals may also be useful, with many, including polyphenols, curcumin, quercetin, soy isoflavones, lycopene and resveratrol shown to reverse epigenetic modifications, often acting on more than one class of epigenetic modification.^281^

## Biologics

### TGF-β antibodies

A number of TGF-β neutralising antibodies have been developed and tested in a range of conditions including OA. Neutralisation of TGF-β may be a powerful therapy that interrupts the positive-feedback loop between this cytokine and myofibroblasts,^94^ and could potentially lead to the resolution of active arthrofibrosis. Several TGF-β neutralising antibodies as well as receptor blocking antibodies have been developed and have passed early clinical trials for fibrotic diseases and cancer.^94^

### IL-1 antibodies and IL receptor antagonists

IL-1 antibodies such as Rilonacept bind to and inactivate IL-1. In addition, IL-1 receptor antagonists (RA) such as anakinra bind to IL-1 receptors, blocking IL-1 from binding, and have been used successfully to prevent arthrofibrosis in small studies^282,283^ and fibrosis of organs.^98,284^ These results suggest that IL-1 is an important player in fibrogenesis, however, further research is needed to investigate the efficacy of anakinra and similar products in preventing or treating arthrofibrosis.

### TNF-α antibodies

TNF-α antibodies have been shown to reduce lung fibrosis in mice^173,285^ and mice lacking TNF-α signalling pathways are protected from lung fibrosis;^170^ however, the use of TNF-α antibodies in fibrogenic diseases has produced contradictory results.^285^ Blocking a single key inflammatory cytokine such as TNF-α can block the cascade of other inflammatory cytokines, including IL-1β and IL-6,^158^ together with the resulting tissue damage and ROS.^285^ This effect, combined with an expected reduction in TGF-β, TGF-β receptors and collagen type I with TNF-α blockade,^286^ suggests that TNF-α antibody treatment may be useful for treating arthrofibrosis. Verjee et al.^287^ demonstrated that TNF-α antibodies inhibited the contractions of myofibroblasts taken from patients with Dupuytren’s disease, which involves progressive fibrosis of the palm. A rat model of fatty liver disease showed that TNF-α antibody treatment reduced inflammation and fibrosis, as well as serum TGF-β in experimental models.^285,288^

In support of this, one patient on the patient website KNEEguru^241^ reported that TNF-α antibody treatment was effective for managing the pain associated with active arthrofibrosis. TNF-α induces peripheral pain sensitisation^56,187,289^ so it is expected that TNF-α antibodies will assist in pain management. However, the usefulness TNF-α antibody therapy for managing arthrofibrosis in the wider patient community is unknown. TNF-α has pleiotropic effects, and its role in organ fibrosis remains controversial.^285,286^ In addition, it may have different effects in different organs.^285^ The importance of TNF-α in established fibrosis remains to be clarified, and further research is required to understand the effectiveness of TNF-α antibodies as a therapeutic agent for treating fibrosis.

For a review of potential pharmacological therapies to treat fibrosis see Nanthakumar et al.^17^

## Mesenchymal stem cells

Mesenchymal stem cells (MSCs) are able to home in on injured tissue and differentiate into different tissue types.^290^ They modulate the immune system by altering the activation and proliferation of immune cells, and are being tested in clinical trials for the treatment of lung fibrosis.^174,291,292^

Some studies have reported positive results using MSCs to treat fibrosis of organs in mice, however, MSC treatment remains controversial. Bone marrow-derived MSCs transform into myofibroblasts in rats^293^ and organ-resident MSC-like cells have been shown by genetic lineage tracing to transform into myofibroblasts and contribute to fibrosis progression in mice.^107^ Mice with liver fibrosis treated with bone marrow-derived human MSCs showed that some of the donated cells appeared to differentiate into myofibroblasts in the liver.^294,295^ It is not known how closely these cells resemble the MSC lineages used in in vitro studies. Nonetheless, the few studies of MSCs in humans to date have not demonstrated worsening fibrosis.^296,297^

It is known that MSCs can contribute to the growth of tumours,^290^ but a recent small phase I clinical trial of MSCs in the treatment of pulmonary fibrosis reported no adverse safety outcomes.^292^ Different preparations of MSCs differ in their efficacy, perhaps because of variation in their expression of anti-inflammatory genes.^298^ The age and origin of MSCs may affect the outcome of fibrosis therapy, as MSCs from different lineages express different proteins that affect their therapeutic potential.^299^ MSCs from the IFP of patients with OA appear to inhibit the production of inflammatory cytokines in vitro.^300^

However, MSCs from the IFP are also capable of differentiating into fibroblasts^25^ in a similar wasy to MSCs from bone marrow.^113^ Furthermore, MSCs from the synovial membrane are positive for the TGF-β receptor CD 105,^299^ raising the possibility that these cells may induce fibrosis under inflammatory conditions. Indeed, TGF-β is understood to be a key factor that recruits MSCs to damaged tissue, and the demonstrated differentiation of MSCs into myofibroblasts^94^ suggests that MSCs are often involved in the pathology of fibrosis.

## Diet

A number of dietary additions may assist those about to undergo surgery to avoid arthrofibrosis, or reduce symptoms in those with an existing condition, although data is lacking. A diet rich in omega 3 fatty acids is recommended for inflammatory conditions (see above in Resolvins). Capsaicin (found in chilli and peppers) and sulphoraphane (found in cruciferous vegetables) have been demonstrated to reverse differentiation of myofibroblasts in vivo. Sulphoraphane has an anti-fibrosis effect via the activation of nuclear factor erythroid 2-related 2, which may be important in preventing fibroblast differentiation.^301^ A diet high in resistant fibre is also likely to be beneficial for preventing inflammation and fibrosis. Gut bacteria produce short-chain fatty acids from non-digestible dietary fibre, which counter inflammation and suppress the cleavage of protease caspase-1 and secretion of IL-18.^302^

In addition, consumption of soy products may help reduce the levels of inflammatory cytokines. The breakdown products of soy isoflavones, daidzein and genistein, are known to be antioxidant and anti-inflammatory compounds, but their usefulness in treating arthrofibrosis has had only limited testing in non-human animals. Liu et al.^303^ found that in rabbits the topical application of daidzein to exposed tissue during surgery reduced fibroblast density, collagen formation and adhesions. Daidzein reduced ROS and levels of TGF-β and when given subcutaneously to rats, reducing lung fibrosis.^150^

Some of the dietary compounds mentioned above also change epigenetic modifications, and were recently reviewed by Khan et al.^281^

Vitamin D is required for immune system homoeostasis, reducing TGF-β, suppressing the Th17 profile, and supporting regulatory T cells that suppress autoreactive T cells.^304^ Vitamin D deficiency is correlated with fibrosis of the liver and vitamin D3 inhibits the production of collagen type 1 in the liver^305^ and in the lung.^306^ This suggests that this important vitamin may be a useful anti-fibrotic agent.^103^

Fibrosis may be promoted by a reduced dietary intake of potassium (K^+^) and low serum K^+^ levels were associated with liver fibrosis.^307^ K^+^ efflux from cells can result from cellular damage and the release of adenosine triphosphate.^308^ Upregulation of the intermediate/small-conductance Ca^2+^-activated K^+^ channel alters the membrane potential of cells and promotes fibrogenesis, with effects including higher levels of Ca^2+^ entry into cells and expression of Ca^2+^-dependant growth factor genes, cyclins and kinases involved in cell division.^309^ High-intracellular levels of Ca^2+^ are associated with cardiac fibrosis^202^and KCa3.1 silencing in animal models of renal fibrosis decreased the numbers of myofibroblasts and attenuated the development of fibrosis.^310^ This result appears to be mediated via the Smad2/3 pathway, since KCa3.1 blockade reduced levels of TGF-β1, and TGF-β1 receptor II.^311^

The loss of intracellular K^+^ also activates NLRP3 inflammasomes, which are known to promote fibrosis. Sun et al. found that low levels of K^+^ promotes vascular calcification and osteogenic differentiation.^312^ This may have implications for the calcification that sometimes occurs within ECM in arthrofibrosis.

Intermittent fasting has profound positive effects on many health measures and can improve functional outcomes for many diseases.^313^ Animal research shows that intermittent fasting is protective against fibrosis of organs,^314^ and it is also known suppress inflammation^313,315^ and downregulate the inflammatory cytokines IL-1, IL-6 and TNF-α in humans.^316^ Intermittent fasting suppresses the activity of NLRP3 inflammasomes,^313^ providing what appears to be a cumulative anti-fibrotic effect. Intermittent fasting can be approached in a number of ways, including restricting calorie intake 2 days of the week, restricting food intake to 8 h or less per day, and periods of 1 or 2 days in which there is little or no calorie intake on a recurring basis.^313^ Nutrient depletion prior to surgery can protect against damage from ischaemic conditions.^317^ Intermittent fasting is also known to reduce levels of insulin-like growth factor-1, which promotes the survival of myofibroblasts in liver fibrosis.^252^

Intermittent fasting may also trigger the amino acid limitation response, which alters immune function by regulating T- and B-cell proliferation, activation and differentiation.^318^ The amino acid l-proline is required for fibrosis, as it is a necessary pre-curser of collagen.^319^ l-proline production can be upregulated by arginine metabolism, resulting in macrophage switching and promoting Th2 cells and fibrosis.^319^

## Other treatments

Recent research suggests that injections of collagenase, a proteolytic bacterial enzyme that specifically breaks down collagen, can increase ROM in arthrofibrosis of the shoulder^320,321^ and in animal models of arthrofibrosis of the knee, however, further research is needed to address concerns relating to degradation of articular cartilage, ligaments and tendons.^322^ In addition, the signalling effects of collagen fragments created by cleavage also needs to be considered, as some of these fragment have biological activity.^104^ However, collagenase has been approved for Dupuytren disease, a fibroproliferative disease of the palm.^323^ Collagenase in slow release nanocapsules have been developed and tested in an animal model of skin fibrosis, showing sustained release over 10 days.^324^ This could lower the number of doses required.

Soft tissue mobilisation techniques using tools designed to exert shear force, break down ECM and stimulate blood flow have been developed, and have several trademark names including ASTYM and the Graston Technique. These tools can be applied to the joint and may assist recovery from arthrofibrosis^32,325,326^ and tendon damage,^327^ particularly before the ECM matures. Some patients on the website KNEEguru report good results,^241^ however, there are few large well-controlled trials, and more research is needed to determine how effective these methods are.^326^

Interventions that increase tissue vascularisation may slow the progression of fibrotic processes.^13^ A small study involving three patients undergoing revision TKR for arthrofibrosis suggested that low dose irradiation prior to surgery may result in improved ROM.^30^

## Patient perspectives

The experiences of arthrofibrosis patients are seldom heard in the scientific literature, and can provide useful insights into the condition. Arthrofibrosis patients on the website KNEEguru^241^ show that there are a group of patients with active arthrofibrosis, for whom surgical intervention to lyse ECM has proven to be detrimental. Another group may have had initially detrimental results from surgery, however, subsequent surgery led to important improvements in symptoms. The reasons for these significantly different outcomes are not clear, but may include the level of inflammation, the time between surgeries, surgical technique, rehabilitation protocols and individual predisposition through genetic or other factors. Unfortunately, as with research publications, these forums are largely silent on the long-term outcomes for patients with permanent active arthrofibrosis.

Many patients believe that overly aggressive exercise rehabilitation soon after surgery was detrimental to their recovery, and strongly advocate that arthrofibrosis sufferers “listen to their knee”, and do not push too hard to fit in with exercise regimes and expected recovery timetables. As mentioned previously, this fits with views expressed recently that rehabilitation should be progressed conservatively, and based on the inflammatory response it provokes.^12,328^

One patient provides a useful case study into the causes and types of arthrofibrosis. Following bilateral TKR a 45-year-old female immediately began intensive post-operative use of CPM in addition to exercise rehabilitation. Initially the right knee had restricted ROM of around 70°. After several months of intensive daily CPM use the knee regained a functional amount of flexion and CPM use was discontinued. The ROM on the left knee was initially 105°, but at 2 weeks post-surgery a minor forced bending on a CPM lacking digital control likely caused internal bleeding, and the knee immediately lost ROM, with maximum flexion falling to 70°. Intensive CPM use gradually increased flexion, however, the knee remained painful and intensive CPM use was required to maintain functional ROM.

After 5 and half months the arthrofibrosis resolved, and the knee became fully functional without pain and with stable active flexion of about 110°. However, 4 weeks after full resolution of arthrofibrosis the patient became ill with suspected influenza. Active arthrofibrosis spontaneously and permanently returned in the left knee, with pain and difficulty maintaining active flexion of 80°.

Several factors are significant in this history. Firstly, the patient has one knee with residual arthrofibrosis (resolved but stiff), while the other knee has active arthrofibrosis, triggered initially by internal bleeding shortly after surgery. Vascular damage is known to promote fibroblast activation.^329^ Secondly, after the resolution of early symptoms influenza appears to have triggered the permanent return of active arthrofibrosis. Many of the inflammatory cytokines that are produced in response to influenza, particularly IL-1, IL-6 and TNF-α^330,331^ are known to cause the differentiation of fibroblasts into myofibroblasts^103^ and are important cytokines in arthrofibrosis (see above). In addition, influenza directly activates NLRP3 inflammasomes,^332,333^ which are implicated in fibrosis. Influenza also activates biologically inactive TGF-β,^146,331^ the primary driver of fibrosis. This suggests that this virus is a significant risk factor for arthrofibrosis, with the risk potentially higher for post-operative patients and those with residual fibrosis.

## Conclusion and future directions

Arthrofibrosis is a fibrotic disease caused by excessive myofibroblast proliferation with defective apoptosis, primarily induced by dysregulated TGF-β signalling. Once established these factors and others, including extensive collagen cross-linking, create a complex web of positive feedback processes that establish a new pathological homoeostasis that maintains excessive ECM accumulation and low-grade inflammation. These processes are initiated by chronic low-grade or acute inflammatory conditions or events.

Surgical lysis and removal of ECM, and MUA, remain the primary treatments for arthrofibrosis. The surgical removal of ECM can be beneficial, not only because it removes the physical stress and restriction to ROM but also because it removes pro-fibrotic mediators bound to the ECM that can fuel the cycle of ECM formation. However, the potential benefits of surgery must be weighed against the risks, which include increased dysregulation of fibrogenesis in response to the surgical injury. This can result in the rapid return of arthrofibrosis with increased severity of symptoms. MUA also carries significant risks such as fractured bones, torn tendons and damaged prostheses and can increase symptom severity.

The development of a minimally invasive diagnostic tool kit that assesses the levels of cytokines, collagen fragments and other mediators of fibrosis in serum and synovial fluid may help to predict which patients are at greatest risk of post-operative arthrofibrosis. This research could provide relatively fast results that may help prevent permanent disability for thousands. Patients found to be at risk could receive anti-fibrotic therapies and intensive monitoring by a rheumatologist to control inflammation.

Early intervention to prevent fibrosis is likely to be important,^14^ halting the process before extensive epigenetic modifications occur and a significant amount of ECM has formed and become strongly cross-linked. This could potentially stop the pathological cascade of dysregulation and positive feedback that results in permanent active fibrosis,^214^ while also preventing damage to joint structures such as cartilage and ligaments that occurs with tissue contraction and altered biomechanics.

Future research should investigate the mechanism of potentiation (memory) from previous surgeries and injuries. Elucidation of this mechanism may indicate the best timing and most appropriate treatment targets to prevent post-operative arthrofibrosis. Anti-fibrotic coatings on surgical implants may prove useful in preventing arthrofibrosis.^248^ In addition, the lack of an effective therapeutic agent to halt or reverse fibrosis after it develops remains an issue of enormous importance for arthrofibrosis and fibrosis of organs. Recent research shows that it is possible to reverse fibrosis but it is still not understood how this occurs.

Until these research priorities are addressed risk factors such as many previous surgeries, pre-existing stiffness or inflammation, early onset OA, childhood adversity and female gender should be considered prior to surgery. In addition, a conservative approach to rehabilitation exercises is recommended with adjustments made according to how the joint responds. Aspirin provides a useful means to both reduce inflammation and induce the production of resolvins, and should be used in preference to other NSAIDS where possible. Low-dose aspirin can be effective, particularly if taken with omega 3 fatty acids. Other aspects of diet and nutrition should also be considered. CPM may be useful for minimising joint contractions, however, research into the use of CPM to treat arthrofibrosis is lacking.

The different disorders that are diagnosed as arthrofibrosis need to be clarified and defined. This includes residual arthrofibrosis with a stiff joint, and active arthrofibrosis in which inflammatory processes and ECM formation are continuing. Attention should be focused on assessing levels of pain, inflammation and functional scores. It is likely that patients with active arthrofibrosis have a higher risk of the return of aggressive arthrofibrosis following surgical intervention or MUA, compared to those with residual arthrofibrosis.

The IFP and pouches within the knee provide relatively contained spaces to trap injected therapeutic agents. This, together with the ability to diagnose arthrofibrosis very early in the process of ECM formation suggests that arthrofibrosis research would be a useful testing ground for fibrosis treatments in general. Likewise, treatments for organ fibrosis are also likely to be useful for treating arthrofibrosis. New therapeutic targets include epigenetic modifications, TGF-β and its downstream signalling, IL-1β, NLRP3 inflammasomes, mast cells, substance P and hypoxia-inducible factor-1. A number of promising therapeutic candidates are currently available, and more are in clinical trials.^17^ A combination of several targets may be needed; however, some existing medications for treating other conditions may prove to be useful.
